# Imaging the dynamics of murine uterine contractions in early pregnancy^†^

**DOI:** 10.1093/biolre/ioae071

**Published:** 2024-05-07

**Authors:** Madeline Dawson, Diana Flores, Lisa Zou, Shivani Anandasenthil, Rohit Mahesh, Olmo Zavala-Romero, Ripla Arora

**Affiliations:** Department of Obstetrics, Gynecology and Reproductive Biology, Michigan State University, East Lansing, Michigan, USA; Institute for Quantitative Health Science and Engineering, Michigan State University, East Lansing, Michigan, USA; Department of Obstetrics, Gynecology and Reproductive Biology, Michigan State University, East Lansing, Michigan, USA; Institute for Quantitative Health Science and Engineering, Michigan State University, East Lansing, Michigan, USA; Department of Obstetrics, Gynecology and Reproductive Biology, Michigan State University, East Lansing, Michigan, USA; Institute for Quantitative Health Science and Engineering, Michigan State University, East Lansing, Michigan, USA; Department of Obstetrics, Gynecology and Reproductive Biology, Michigan State University, East Lansing, Michigan, USA; Institute for Quantitative Health Science and Engineering, Michigan State University, East Lansing, Michigan, USA; Department of Obstetrics, Gynecology and Reproductive Biology, Michigan State University, East Lansing, Michigan, USA; Institute for Quantitative Health Science and Engineering, Michigan State University, East Lansing, Michigan, USA; Department of Scientific Computing, Florida State University, Tallahassee, Florida, USA; Department of Obstetrics, Gynecology and Reproductive Biology, Michigan State University, East Lansing, Michigan, USA; Institute for Quantitative Health Science and Engineering, Michigan State University, East Lansing, Michigan, USA

**Keywords:** uterine contractions, spatiotemporal maps, waveform metrics, pre-implantation uterine contractility, LPAR3

## Abstract

Uterine muscle contractility is essential for reproductive processes including sperm and embryo transport, and during the uterine cycle to remove menstrual effluent. Even still, uterine contractions have primarily been studied in the context of preterm labor. This is partly due to a lack of methods for studying the uterine muscle contractility in the intact organ. Here, we describe an imaging-based method to evaluate mouse uterine contractility of both the longitudinal and circular muscles in the cycling stages and in early pregnancy. By transforming the image-based data into three-dimensional spatiotemporal contractility maps, we calculate waveform characteristics of muscle contractions, including amplitude, frequency, wavelength, and velocity. We report that the native organ is highly contractile during the progesterone-dominant diestrus stage of the cycle when compared to the estrogen-dominant proestrus and estrus stages. We also observed that during the first phase of uterine embryo movement when clustered embryos move toward the middle of the uterine horn, contractions are dynamic and non-uniform between different segments of the uterine horn. In the second phase of embryo movement, contractions are more uniform and rhythmic throughout the uterine horn. Finally, in *Lpar3*^−/−^ uteri, which display faster embryo movement, we observe global and regional increases in contractility. Our method provides a means to understand the wave characteristics of uterine smooth muscle in response to modulators and in genetic mutants. Better understanding uterine contractility in the early pregnancy stages is critical for the advancement of artificial reproductive technologies and a possibility of modulating embryo movement during clinical embryo transfers.

## Teaser

The imaging-based method of ex vivo uterine contractility demonstrates quantitative differences in contractions during estrous cycle, pre-implantation stages of pregnancy, and in LPAR3-deficient mutants.

## Graphical Abstract

**Graphical Abstract:**
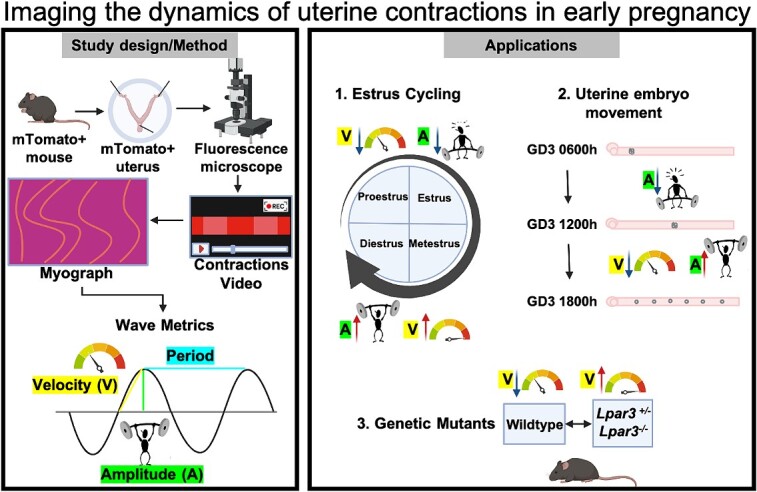

## Introduction

Smooth muscle performs organ-specific functions throughout the body [1]. For instance, smooth muscle in the stomach and intestines aids in food peristalsis and nutrient absorption [2] while smooth muscle in the bladder wall is responsible for the whole organ contraction and relaxation during retention and urination [3]. In regard to the reproductive tract, uterine smooth muscle plays an important role in the movement of eggs to the fundus during ovulation or sperm toward the fallopian tube (oviduct) during fertilization. However, contractions are also key to a woman’s non-pregnant state in discarding menstrual effluent and removing eggs released every cycle. Efforts have been made toward measuring uterine contractions in late-term pregnancy due to the challenges caused by preterm labor. Contractions in the non-pregnant but cycling uterus and the early pregnant uterus differ from the late-term pregnant organ [4]. Thus, imaging methodologies must be designed to capture contractile activity in the non-pregnant and early pregnant uterus.

In all mammals, the uterine smooth muscle is derived from the Mullerian duct (embryonic precursor of the uterus) mesenchyme [5]. In rodents (rats and mice), each Mullerian duct develops into a uterine horn, whereas the two horns fuse to form a single uterine chamber in primates (humans). In both mice and humans, the uterine muscle surrounds the inner epithelium (endometrium) and the mesenchyme (stroma) and comprises of an outer layer of longitudinal muscle and an inner layer of circular muscle. While in most mammals there is a clear separation between the longitudinal and circular muscle layers, this separation is less clear in larger mammals such as humans [5]. Instead, there exists a junctional muscle zone (displays properties of circular muscle) that separates the outer uterine muscle (displays properties of longitudinal muscle) from the inner endometrium [4]. Despite the two distinct muscle layers in mice, a junctional muscle zone was recently reported supporting the utility of mouse as a model to understand the patterns of and the mechanisms regulating uterine contractions [6].

Uterine contractions can be measured using invasive techniques such as intrauterine pressure measurement [7, 8] and electromyography [9] or non-invasive molecular imaging techniques such as three-dimensional (3D) ultrasounds [9] or magnetic resonance imaging [10]. In vivo, pressure recordings and ultrasound can themselves contribute to contractility, and magnetic resonance imaging (MRI) and ultrasounds do not give spatial and quantitative waveform metrics such as amplitude, frequency, and velocity [11–13]. Uterine contractions have been measured by isolating either tissue strips from the uterus [14–17] or transverse uterine slices [12]. Tissue pieces are equilibrated in an organ bath [18] and spontaneous contractions or the effect of agonist induced contractions is estimated. It is critical to note that often, in these methods, spontaneous contractions are measured after a mechanical force is applied using a tension transducer. Limitations of these methods include variability in data generated as different transducer forces will result in different contractile activity that may not reflect spontaneous contractility. Furthermore, separating the uterine muscle from the endometrium or slicing the uterus leads to loss of spatial information [19].

During the estrus cycle, using uterine strips from rat uteri in an organ bath under the effect of a tension transducer, electric field-induced contractility was highest at the estrus stage, and the lowest responses were observed at the diestrus stage [20]. In vivo imaging of the contractions of the rat uteri was attempted by Crane and Martin [21] and generated contrasting results. In this study, three methods were used: a balloon was inserted into the lumen to study intraluminal pressure, electrodes were attached to both the cervical and oviductal ends of the uterus to measure contractions, and laparoscopy was used on anesthetized rats to record the uterus moving in vivo. The use of balloon and electrodes caused rats to lose their cyclicity and thus laparoscopy surgery was used to evaluate contractions during estrous cycle. Qualitatively, total contractile activity was determined to be lowest at proestrus, increased at estrus, and highest during diestrus. The entire mouse uterine horn has also been subjected to a video recording in the non-pregnant state in vitro [14]. Similar to the rat uterus in vivo recordings, this method showed that even in the mouse, diestrus is the most contractile stage. However, when tension transducer was applied to the intact uterine horn and contractions were quantified under mechanical tension, diestrus showed low-frequency and low-amplitude contractions while proestrus showed high-frequency and high-amplitude contractions. These studies highlight conflicting data on which stage of the estrous cycle is most contractile.

An advancement of the whole horn method [22] used pressure readings to calculate amplitude, image-based changes in uterine horn edges to calculate contraction velocity, frequency, and directionality and electrical-induced activity to measure spike potential and burst duration for a proestrus-staged uterus. This method and others have shown that fluid mediated distension increases uterine contractility [22, 23]. Most recently, imaging-based methods have been used to measure both strength and directionality of contractions in the proestrus stage of the mouse uterus using in vivo recordings and spatiotemporal mapping [24]. The limitations of this study are that only the proestrus stage, which displays limited uterine length and lower strength contractions, could be recorded. Further, the use of anesthetics could potentially influence smooth muscle contractions. None of the reported methods permit the study of early stages of pregnancy to evaluate the relationship between uterine contractility and early pregnancy embryo movement.

In all prior studies that used spatiotemporal mapping to examine contractility, the change in diameter between uterine edges was used as a proxy for muscle contraction. Thus, these methods quantify contractions caused by the circular smooth muscle but do not account for longitudinal muscle contractions [14]. On the other hand, when isolating uterine strips, the contractility would depend on the direction in which the strips are loaded into the bath and where the tension transducers are applied to determine whether circular or longitudinal muscle activity was measured [20]. All methods to date fail to report combined contributions of the circular and longitudinal smooth muscle to uterine contractility.

Contractions are critical to embryo movement in the uterus [25]. Thus, there is an urgent need to develop methods to evaluate uterine contractility during early pregnancy. For pregnant mice, oviductal contractions have been quantified by recording changes in the oviductal wall and tracing the movement of the egg/embryo, and directly correlating the movement of the muscle wall with the movement of the egg in the oviductal space [26]. Studies such as these cannot be performed in the uterus as the thickness of the murine smooth muscle layer (~500 μm) prevents optical light from passing through and reaching the uterine lumen where the embryos are residing. Optical coherence tomography has been used to track sperm and oocyte movement in the oviduct [27]; however, this methodology has not been successfully applied to the uterus yet. Here, we discuss an ex vivo imaging-based method to trace uterine contractions during early pregnancy in tomato transgene expressing reporter mice. First, we apply this method to different stages of the estrous cycle and compare our results to previously published data. We then apply our method to pre-implantation stages of pregnancy and compare contractility patterns to our recently published embryo movement patterns [25]. Finally, we apply this method to genetic mouse models with disrupted embryo movement patterns (*Lpar3^−/−^*) [25, 28, 29] and detect global and spatial differences in uterine contractility that explain the differential embryo movement patterns.

## Methods

### Animals

All animal research was carried out under the guidelines of the Michigan State University Institutional Animal Care and Use Committee. CD1 (ICR), wildtype (WT) C57BL/6 J, and *Lpar3^tm1JCh^* (*Lpar3^+/−^* and *Lpar3^−/−^*) mice [29] carrying the Rosa mTmG allele [30] were generated. Mice were maintained on a 12 h light/dark cycle. For non-pregnant studies, the estrus stage was determined using vaginal smear cytology [31]. Dissections were performed at each stage of the estrous cycle: estrus, proestrus, metestrus, and diestrus. For pregnancy studies, adult females aged 6–12 weeks, were mated with fertile WT males to induce pregnancy. The appearance of a vaginal plug was identified as GD0.5 or GD0 1200 h. For CD1 females, uterine dissections were performed on times when embryos are moving in the uterus—GD3 at 0600, 1200, and 1800 h and post-implantation—GD4 at 1200 h. In WT, *Lpar3^+/−^* and *Lpar3^−/−^* (C57BL/6 J background), dissections were performed on GD3 at 0600, 1200, and 1800 h and on GD4 at 1200 h. A minimum of three mice were analyzed for each condition to ensure data reproducibility in independent events.

### Ex vivo, spatiotemporal video recording of the contracting uterus

**Figure 1:**
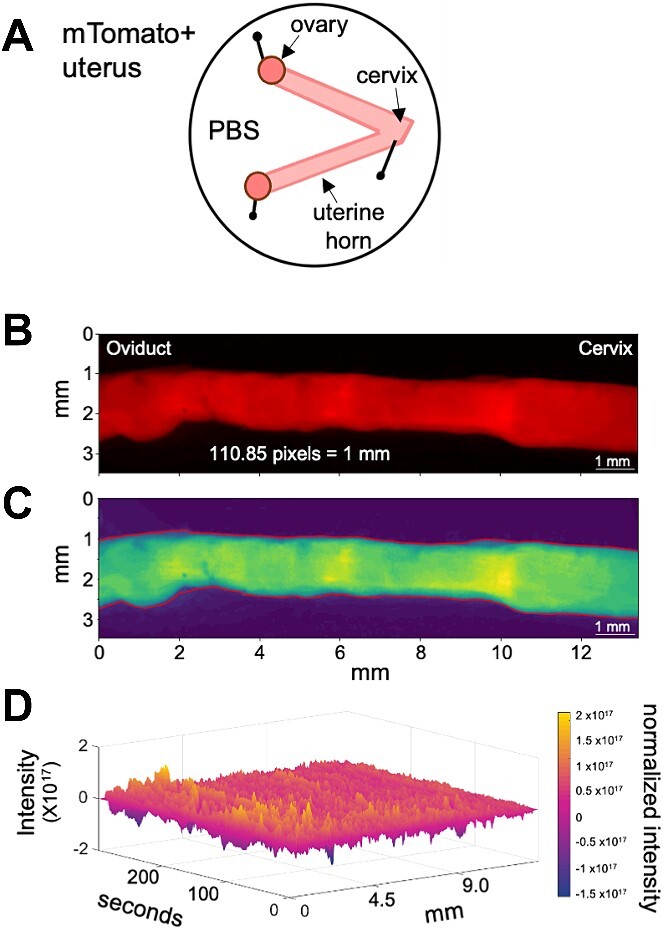
Setup for uterine horn ex vivo imaging and image analysis. (A) Schematic of how the uterine horns are pinned after dissection. (B) Example frame from uterine contraction video displaying tomato positive uterine horn. (C) Smoothened image of (B) showing variation in contraction intensity with blue being the most relaxed and yellow being the most contracting region. (D) Example 3D spatiotemporal plot obtained from image processing with *x*-axis signifying uterine horn distance in pixels or millimeters, *y*-axis signifying frames or time in seconds and *z*-axis in color signifying contraction either as changes in area or changes in tomato intensity.

Immediately after sacrificing the mouse, the uterus was harvested and secured in a plastic petri dish using syringe needles. Three needles were used to hold the uterus in place within the petri dish (one pierced through each ovary and one pierced through the cervix) to mimic the position of the uterus in vivo (Figure 1A). The petri dish containing a pinned uterus immersed in phosphate-buffered saline (PBS) was then placed under a Leica MZ10F fluorescence stereo microscope with a fluorescence filter for Texas Red. Time-lapse uterine contractions were recorded using the LASX software with images captured every 200 ms for 5 min (total 1500 frames). After this initial video was taken, 100 μl of 10 mg/ml solution of salbutamol sulfate prepared in 1:9 ethanol:PBS was added uniformly to stop the uterus from contracting [25]. Another set of images was acquired for 5 min as a non-contracting control. In a separate set of experiments, after the initial video was taken, 100 μl of 60 mm KCl solution (made in Krebs buffer) was added uniformly to the uterus to induce contractility [32]. Another set of images was acquired for 5 min. Next, .lif files from the LASX software were converted to .mp4 files and edited using the virtual dub application (ver 1.9.11). Virtual dub was used to crop .mp4 videos, to get one uterine horn per video, and to threshold tomato intensity and increase the contrast between the uterus and the video background (Figure 1B). Care should be taken to not saturate the tomato intensity as contractile differences rely on changes in tomato intensity along the uterine horn. .mp4 videos were then converted into .avi files using the “Xvid MPEG-4 codec” compression option within virtual dub. Each video produced two .avi files, one for each uterine horn (Supplementary Video 1). These .avi files were then subjected to image analysis to calculate contractility waveform metrics.

### Methodology for extracting waveform metrics from uterine contraction videos

Our methodology aims to identify and characterize two types of contractions observed in videos of the uterine horn (Figure 1B and C). The first type of contraction, which is believed to originate from the circular muscle, is identified by changes in the distance between the upper and lower borders of the uterine horn in the plane perpendicular to the camera's view. For clarity, we will refer to this distance as the “area” since it measures the space between the top and bottom edges of the uterine horn in each image’s column, with the assumption that each pixel has a unit width. The variations in the area metric serve as a proxy for the actual changes in the cross-sectional area of the uterine horn. The second contraction type, hypothesized to originate from a combination of both the longitudinal and circular muscle, is detected through alterations in pixel intensity within the video frames. These intensity changes correlate with tomato reporter density shifts and the number of cells intersected by planes parallel to the camera's viewpoint (Figure 1D). When the tissue contracts, the cell density increases resulting in an increase in the tomato reporter intensity. Regions adjacent to contracting tissue stretch to maintain uterine tissue length, either horizontally or vertically in relation to the camera's Cartesian grid. Cells in the direction of stretch are spread over a larger area. This spreading leads to a reduction in tomato reporter concentration and a corresponding decrease in pixel intensity. Importantly, this alteration in intensity does not depend on the contraction's direction. Therefore, the intensity-based method is responsive to both types of contractions.

Our methodology contains three main steps: identifying the uterine horn in each frame of the videos, estimating the changes in area and intensity through time at each longitudinal position of the uterine horn, and characterizing the contractions detected by estimating the amplitude, frequency, velocity, and wavelength of the contractions.

#### Step 1: uterine horn identification

The initial step in video preprocessing involves automatic detection of the uterine horn's upper and lower boundaries in each frame and column. This task is challenging in areas where the uterine horn is exceedingly thin, as the camera sensor yields minimal intensity gradients at the horn's boundary, making precise boundary determination difficult. Additionally, blood stains in the petri dish introduce further intensity gradients that may confound the detection of the horn's start and end points.

**Figure 2:**
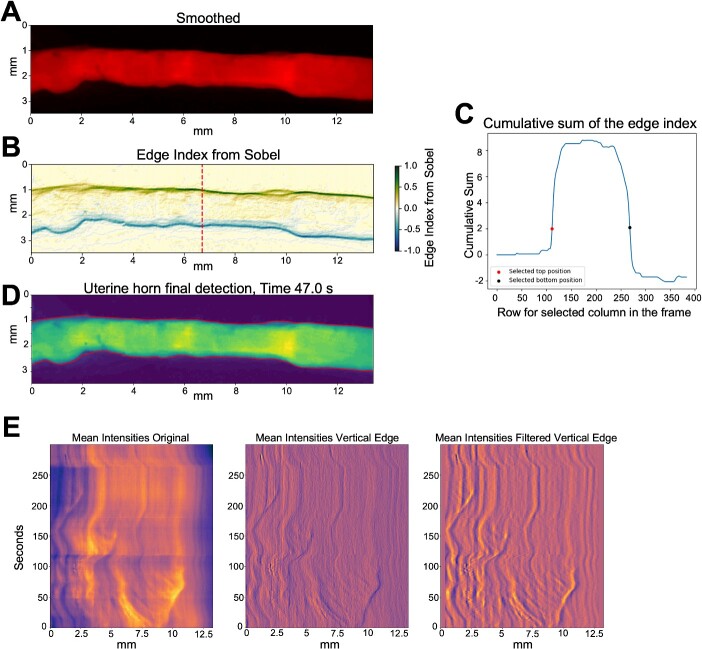
Extracting intensity information from contraction videos. (A) Smoothened image of a single frame of an example uterine contraction video frame. (B) The edge index information obtained through the Sobel filter, (C) corresponding cumulative distribution of the edge index at the mean row (red line) of the analyzed frame, together with the selected locations delineating the limits of the uterus. (D) Automatically obtained limits of the uterus. (E) Mean intensity data obtained from the full video sequence and the post processed filters used to enhance the data.

To delineate the horn's boundaries (Supplementary Video 2), we first apply a horizontal and temporal smoothing process to the videos (Figure 2A), which mitigates any noise artifacts that might interfere with automatic detection. This smoothing is accomplished using a 3-size Gaussian filter in time and a 5-size Gaussian filter in space, implemented via the GaussianBlur function of OpenCV [33]. These filter parameters were manually selected based on a subjective comparison of the original and smoothed video frames. Importantly, this video intensity smoothing is solely for accurate uterine horn boundary selection and is not utilized in subsequent intensity analyses.

Next, we employ the Sobel filter to detect horizontal and vertical edges within each video frame. The Sobel filter's size in both directions is 5 × 5. To give greater weight to vertical edges and generate a single edge index image, vertical edges are weighted according to the formula:


$$ edge\ index={S}_h+2{S}_v $$

where ${S}_h$ is the horizontal Sobel filter and ${S}_v$ is the vertical Sobel filter. Figure 2B illustrates an example of the edge index derived from smoothing a single frame and employing Sobel to primarily detect vertical edges.

The identification of the uterine horn boundary is based on locating the first and last positions where the cumulative sum of the edge index values surpasses a predefined threshold, as illustrated in Figure 2C. This threshold has been manually optimized in our model, and a value of two was found to be effective across all analyzed videos (Figure 2D). As long as the videos have similar intensities, the detected start and end row locations of the uterine horn are not very sensitive to selecting these parameters. To ensure a smooth transition in the identification of the uterine horn boundaries, we apply cubic interpolation to correct any abrupt changes. The interpolation uses knot points spaced at intervals of 1 every 10 columns. While this spacing was constant in our analyses, it can be adjusted for different experimental setups to consider different wave frequencies within the analyzed data.

#### Step 2: area and intensity change estimation

The locations established in Step 1 serve to extract initial and final positions, as well as mean intensity values for each column in each frame of the analyzed videos. We then utilize these values to examine the temporal behavior of the two muscle types via Hovmoller plots [34]. These plots feature the spatially analyzed value (area or mean intensity) on the *x*-axis and time on the *y*-axis. To identify space changes, we employ a 5-size Sobel kernel filter, striking a balance between filtering minor spatial and temporal variations and detecting changes in each field. Finally, we filter the low-frequency signal in space and then apply a low-pass filter to remove noise again. Figure 2E shows the result of this step of a complete video for the intensity variable. For further details, see Supplementary Methods.

#### Step 3: contraction characterization

**Figure 3:**
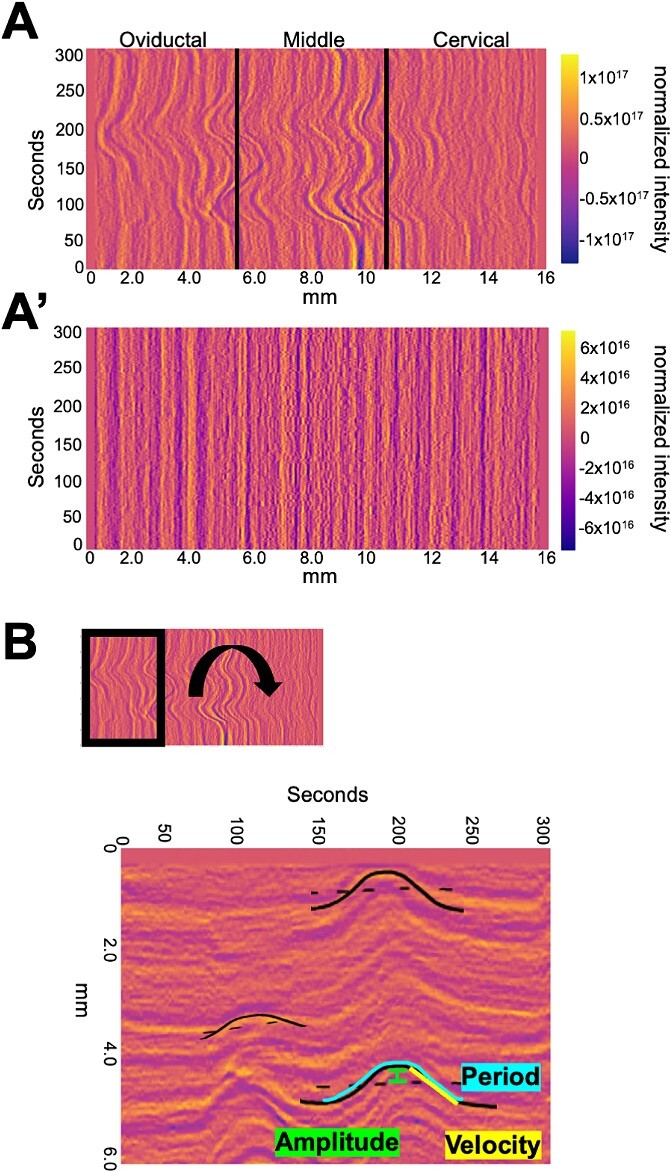
Calculating waveform metrics. (A) Example spatiotemporal plot of mean intensities showing contractile wave activity in three segments of the uterine horn—oviductal, middle, and cervical. (A’) Uterine horn in A treated with salbutamol showing no contractile activity. (B) Example of how waveform metrics such as amplitude, velocity, and period are calculated from the waves in the spatiotemporal area or intensity plots.

Intensity- or area-based output plots (Figure 3A) were used to quantify uterine horn contractions. We compared intensity plots from a contracting uterus (Figure 3A) and corresponding salbutamol-treated non-contracting uterus (Figure 3A**’**) to ensure that changes observed were indicative of muscle movement and not changes in uterine shape along the length of the horn. Contraction characterization is achieved using a technique commonly employed in meteorology, where we identify lines in the Hovmoller plot [34] and correlate these with the properties of a moving wave. Amplitude and period were calculated as from the distance (pixels) − time (frames) graph by manually selecting waves as outlined in Figure 3B (see Supplementary Figure 1 and Supplementary Methods). Time was converted from frames to seconds, with 5 frames per second resulting in 1500 frames or 300 s. Distance was calculated in millimeters from pixels where 110.85 pixels = 1 mm. Frequency was calculated as the inverse of period. The wave's velocity is determined by the line's slope, with flatter lines indicating faster speeds (see Supplementary Figure 1 and Supplementary Methods). The wave's direction is dictated by the slope of the line, with positive and negative slopes signifying rightward (toward cervix) and leftward (toward oviduct) movement, respectively. Using the slope of the line we did observe waves traveling toward both the oviduct and the cervix, however for this study, we focused on evaluating the spatial distribution of contractions and did not focus on the directionality of contractions. Wavelength is calculated as velocity divided by frequency. For segment analysis, each “mean intensities” graph was sectioned into thirds using lines perpendicular to the *x*-axis at each third of the *x*-axis, effectively splitting the graph into oviductal, middle, and cervical segments (Figure 3A). Three waves were selected at random from each segment and wave function analysis was performed on a total of nine waves per uterine horn. These waveform metrics were then used to compare contractions between different experimental groups.

### Whole-mount immunofluorescence

Whole-mount immunofluorescence staining for WT, *Lpar3^+/−^*, and *Lpar3^−/−^* uteri was performed as described previously [35]. Uteri were fixed in DMSO:methanol (1:4) after dissection. To stain the uteri, they were rehydrated for 15 min in 1:1, methanol: PBST (Phosphate Buffer Saline (PBS), 1% Triton X-100) solution, followed by a 15 min wash in 100% PBST solution before incubation. Samples were incubated with Hoechst (B2261, Sigma-Aldrich, St. Louis, MO, USA) diluted in PBST (1:500) for two nights at 4°C. The uteri were then washed once for 15 min and three times for 45 min each, using PBST. Next, the uteri were stretched in 100% methanol, followed by 30 min dehydration in 100% methanol, an overnight incubation in 3% H_2_O_2_ solution diluted in methanol, and a final dehydration step for 60 min in 100% methanol. Finally, samples were cleared using a 1:2 mixture of benzyl alcohol:benzyl benzoate (108006, B6630, Sigma-Aldrich, St. Louis, MO, USA).

### Confocal microscopy

Confocal imagining procedures were done as previously described [25]. Stained uteri were imaged using a Leica TCS SP8 X Confocal Laser Scanning Microscope System with white-light laser, using a 10× air objective. For each uterine horn, z-stacks were generated with a 7.0 μm increment, and tiled scans were set up to image the entire length and depth of the uterine horn [34]. Images were merged using Leica software LASX version 3.5.5.

### Image analysis for embryo location

Image analysis was done using commercial software Imaris v9.2.1 (Bitplane, Zurich, Switzerland). Embryo location was assessed as described previously [25]. Briefly, confocal LIF files were imported into the Surpass mode of Imaris and Surface module 3D renderings were used to create structures for the oviductal-uterine junctions, embryos, and horns. The 3D Cartesian coordinates of each surface’s center were identified and stored using the measurement module. The distance between the oviductal-uterine junction and an embryo (OE), the distance between adjacent embryos (EE), and the horn length was calculated using the orthogonal projection onto the XY plane. All distances were normalized to the length of their respective uterine horn. Horns with less than three embryos were excluded from the analysis. These distances were used to map the location of the embryos relative to the length of the uterine horn. The uterine horn was divided into three equally spaced segments—closest to the oviduct, middle, and closest to the cervix. These segments were quantified for the percentage of embryos present in each section. Embryos in the oviductal region close to the oviductal-uterine junction were accounted for in the first oviductal segment.

### Statistical analysis

Data groups were compared using the Mann–Whitney test (unpaired experimental design, non-parametric test, compare ranks) with resulting two-tailed *P*-values. *P*-values <0.05 were considered significant. Statistical Analysis was performed using GraphPad Prism 8.2.1.

Outliers were identified and removed using the ROUT (robust regression and outlier) method [36] with Q, the maximum desired false discovery rate, set at 1%. The ROUT method uses n outlier detection method, based on the false discovery rate, to choose outliers that are outside the prediction of the model. Statistical comparisons and *P*-values for different comparisons are presented as supplementary tables accompanying the figures.

## Results

### Ex vivo method to measure and compare uterine contractility

Typically, uterine horns vary in length based on genetic background (mixed background CD1/ICR mice have longer uterine horns than C57Bl6 mice). Further, during pregnancy, there is proliferation and increase in the length of the uterine tissue [37]. Thus, it is key to measure contractility in a dynamic range of uterine horn lengths and different pregnant and non-pregnant stages. Our method preserves the structure of the uterine horn and relative orientation in the in vivo setting. In vivo, the ovaries are attached to the kidneys (fixed structures) and the cervix is attached to the body wall. We mimic this configuration in the petri dish by pinning the oviductal and cervical ends while allowing spontaneous contractility to occur freely in the body of the uterus.

### Measurement of contractile behavior using change in area vs change in tomato intensity

Contractility can be measured based on the movement of the edges of the smooth muscle that surrounds an epithelial lumen. This method has been employed to measure contractility in the oviduct [26, 38] and the non-pregnant uterus [22, 24]. Further, since the uterine cells express tomato reporter, increase in tomato intensity highlights a contracting region while loss of tomato intensity suggests muscle relaxation. Uterine smooth muscle is comprised of an outer longitudinal layer and an inner circular layer. Considering that we are using whole tissue two-dimensional images for estimating contractility, changes in tomato intensity likely reflect combined effects from both longitudinal and underlying circular smooth muscle layers (Figures 1–3).

**Figure 4:**
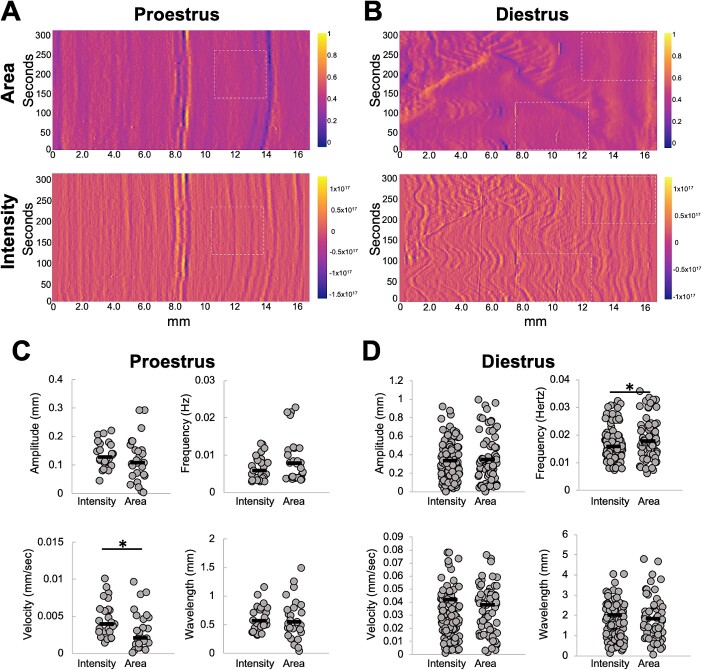
Metrics obtained from area vs intensity spatiotemporal plots. Normalized differences in area (top) vs intensity (bottom) in a proestrus (A) and diestrus (B) stage uterine horn. Amplitude, frequency, velocity, and wavelength calculations from area and intensity plots from proestrus uterine horn in A (C) and diestrus uterine horn in B (D). For this comparison, all waves detected visually in both stages were marked and quantified. White dotted rectangles highlight regions in intensity plots where contractions are observed, but corresponding regions in area plots do not show contractions. *n* = 3 uterine horns for each stage. ^*^  *P* < 0.05. Black lines indicate medians.

We first compared data generated from the area plots to the intensity plots across the proestrus and diestrus stages (Figure 4A–D). Our analysis suggested that area plots often had regions where contractility was not apparent, although in the corresponding regions of the intensity plots, contractility was observed. We wanted to spatially analyze contractility during preimplantation stages; thus, we used contractility measurements originating from the intensity plots and not the area plots. When comparing values of different waveform metrics, we observed that the range of amplitude, velocity, frequency, and wavelength were relatively similar across both area and intensity plots. We also observed that all of these metrics were significantly higher at the diestrus stage (Figure 4B and D) compared to the proestrus stage (Figure 4A and C). These data suggest that spontaneous contractions are stronger and more frequent in the diestrus stage when compared to the proestrus stage (Supplementary Table 1).

### Diestrus-stage uteri display maximum contractility

**Figure 5:**
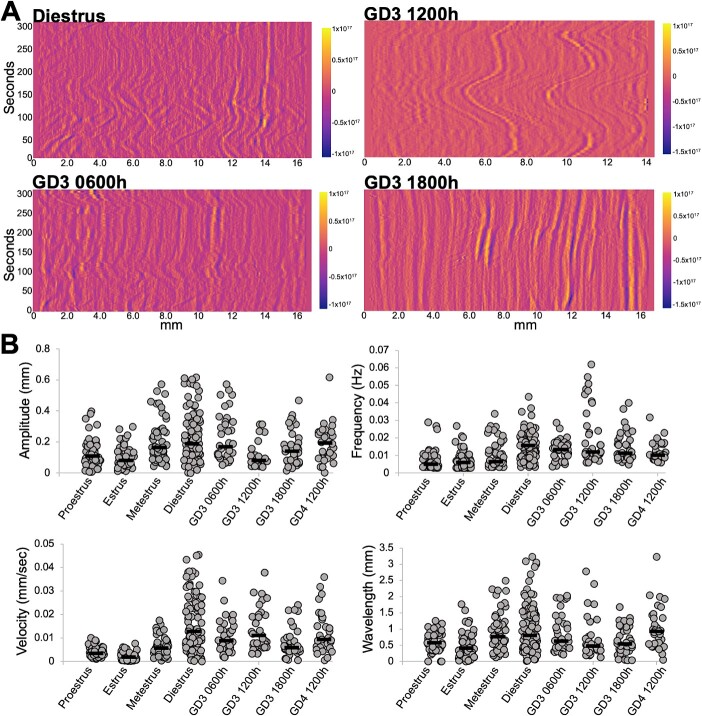
Contractility in pre-implantation stage uteri is closest to the non-pregnant diestrus stage. (A) Example spatiotemporal plots for uterine contractility from diestrus, GD3 0600, 1200, and 1800 h stages. (B) Amplitude, frequency, velocity and wavelength calculations from intensity plots of different uteri. Proestrus (*n* = 3 mice, 6 uterine horns); estrus (*n* = 3 mice, 6 uterine horns); metestrus (*n* = 3 mice, 6 uterine horns); diestrus (*n* = 6 mice, 9 uterine horns); GD3 0600 h (*n* = 4 mice, 4 uterine horns); GD3 1200 h (*n* = 4 mice, 4 uterine horns); GD3 1800 h (*n* = 3 mice, 4 uterine horns); and GD4 1200 h (*n* = 4 mice, 4 uterine horns). For this comparison, nine waves per plot distributed throughout the uterine horn were marked and quantified. For statistics refer to Supplementary Tables 2 and 4. Black lines indicate medians.

To date spatial contractility analysis for the entire uterine horn has only been performed on the stages of estrous cycle in non-pregnant mice. To compare our method to published methods, we first applied our method to the different stages of the estrous cycle (Supplementary Figure 2). We observed that diestrus staged uteri display contractions of the highest frequency (0.016 Hz), and velocity (12.8 μm/s) compared to each of the other estrous phases (*P* < 0.05 or *P* < 0.0001). Diestrus also showed the highest amplitude (189.4 μm) and wavelength (802.9 μm) that was significantly different from proestrus and estrus (*P* < 0.0001) (Figure 5A and B, Supplementary Table 2). There were similarities between estrus and proestrus stages reflecting estrogen dominance (median values of waveform metrics are reported in Supplementary Table 2). In the estrus, proestrus, and metestrus stages, low-velocity and low-frequency waves were observed largely without a directionality bias toward the oviduct or the cervix (Supplementary Figure 2). In the estrus stage, we observed that two out of six uterine horns showed a single contractile wave moving toward the oviduct (Supplementary Video 3, Supplementary Figure 2); however, in the remaining four out of six horns, we did not observe a directionality bias toward either end of the uterus (Supplementary Video 4, Supplementary Figure 2). For the diestrus stage (Supplementary Video 1), we observed several contractile waves of wavelength and velocity higher than other estrous cycle stages (Supplementary Figure 2). Waves originated at multiple places in the uterine horn. However, we did not discern any bias toward the oviductal or cervical end.

### Potassium chloride alters velocity and wavelength waveform metrics

Next, we used potassium chloride to induce changes in uterine contractility in metestrus-staged uteri. We recorded contractility for 5 min, added 60 mm KCl [32] and recorded contractility for an additional 5 min (Supplementary Figure 3A). We then compared the waveform metrics: amplitude, frequency, and velocity and wavelength right before adding KCl (150–300 s) and immediately after the addition of 60 mm KCl (300–450 s). We observed that with the addition of KCl, wavelength and velocity of waves showed a significant increase (Supplementary Figure 3B). Increase in these metrics was transient and returned to baseline over the next 150 s (450–600 s). Amplitude and frequency remained the same despite addition of KCl (Supplementary Table 3).

### Pre-implantation pregnancy contractility is most similar to the non-pregnant diestrus stage

The biggest advantage of our method is the ability to assess spatiotemporal uterine contractility in stages of early pregnancy. Pre-implantation, gestational day (GD) 3 of mouse pregnancy displayed contractility most comparable to the diestrus stage of the cycle (Figure 5A and B, Supplementary Table 4). This is likely due to the progesterone dominance of both GD3 and diestrus stage. GD3 is also the stage where blastocyst- staged embryos enter the uterine horn (GD3 0000 h), move as clusters to the center of the uterine horn (GD3 0300–1200 h), and then scatter and space out through the uterine horn (GD3 1200–1800 h) before they attach on GD4 (0000 h) [25]. We have previously shown that clustered embryo movement is reliant on uterine contractions whereas embryo scattering is independent of uterine contractions. Thus, we assessed contractility corresponding to different phases of embryo movement. We observed that contractions during the early stages of clustered embryo movement (GD3 0600 h) display a higher amplitude (median 171.4 μm) reflecting active movement of embryos at GD3 0600 h. At GD3 1200 h, the amplitude drops drastically (median 81.2 μm) likely because at this time embryos are held in the center of the uterine horn. Intriguingly, both GD3 0600 and GD3 1200 h display a higher velocity of uterine contractions (8.9 and 11.1 μm/s, respectively). Velocity of contractions drops during the second phase of embryo movement at GD3 1800 h (median 5.9 μm/s) concurrent with embryo scattering. Similar to diestrus, the frequency of uterine contractions stays high throughout pre-implantation embryo movement stages (>0.01 Hz) suggesting that this waveform metric may be responsive to presence of objects inside the uterine horn (eggs in diestrus and embryos in pre-implantation-staged uteri). Wavelength on different GD3 time points is comparable to each other but lower than diestrus. Wavelength may be a factor dependent on the diameter of the uterine lumen and may also respond to presence of embryos in the uterine lumen. As the embryos implant and begin to form an implantation chamber at GD4 1200 h, the waveform metrics are comparable to the diestrus stage, and the contractions are more rhythmic throughout the whole horn and spatial variations are not evident.

### Application of contractility method to explain embryo movement patterns in a mouse model with genetic perturbation

**Figure 6:**
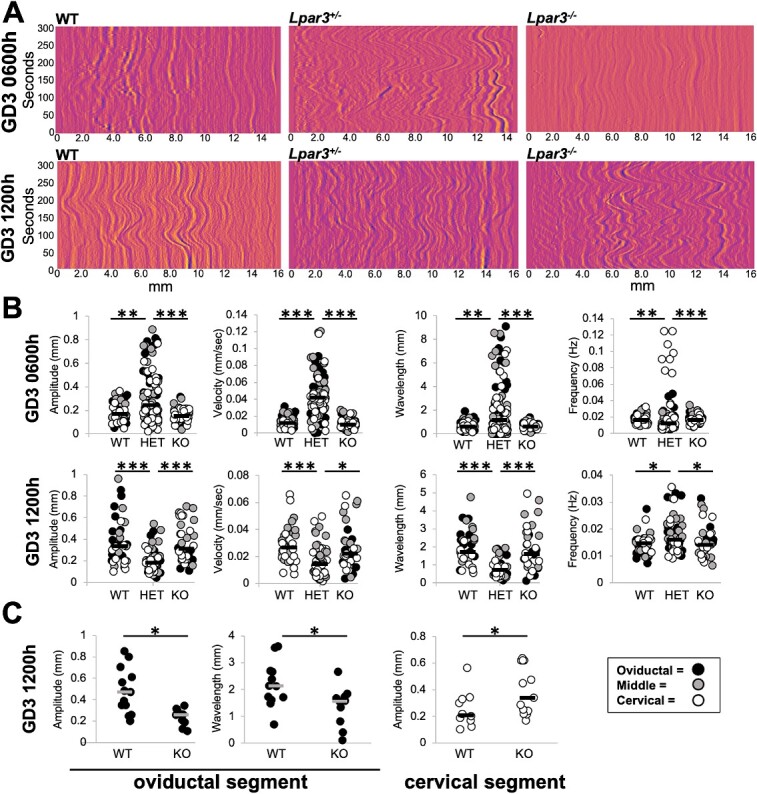
LPAR3-deficient uteri display increased contractility in the preimplantation stages**.** (A) Example spatiotemporal plots for uterine contractility from GD3 0600 h and GD3 1200 h for WT, *Lpar3^+/−^* (HET), and *Lpar3^−/−^* (KO) uteri. (B) Amplitude, frequency, velocity, and wavelength calculations from intensity plots of uteri from WT, *Lpar3^+/−^*, and *Lpar3^−/−^* uteri. Increased global contractility is observed in *Lpar3^+/−^* uteri compared to WT and *Lpar3^−/−^* uteri. (C) When separated by segment, *Lpar3^−/−^* uteri display decreased contraction amplitude and increased wavelength in the oviductal segment but display increased contraction amplitude in the cervical segment. GD3 0600 h WT (*n* = 3 mice, 5 uterine horns); *Lpar3^+/−^* (*n* = 4 mice, 8 uterine horns); *Lpar3^−/−^* (*n* = 3 mice, 6 uterine horns). GD3 1200 h WT (*n* = 3 mice, 4 uterine horns); *Lpar3^+/−^* (*n* = 3 mice, 6 uterine horns); *Lpar3^−/−^* (*n* = 3 mice, 4 uterine horns). For this comparison, nine waves per plot distributed throughout the uterine horn were marked and quantified. ^*^P < 0.05; ^*^^*^  *P* < 0.001; ^*^^*^^*^  *P* < 0.0001. Black and white lines indicate medians.

Lysophosphatidic acid (LPA) signals through its receptor LPAR3 in the uterus. This signaling is key for embryo implantation and affects both embryo movement [25, 29] as well as uterine contractility [30]. However, contractility in LPAR3 mutants was measured using uterine strips and in response to applied tension. Thus, we evaluated spontaneous uterine contractility in WT, *Lpar3^+/−^* and *Lpar3^−/−^* uteri using our method. Intriguingly, we observed that when measured globally, metrics for uterine contractility were comparable between WT and *Lpar3^−/−^* uteri and instead the *Lpar3^+/−^* uteri displayed differential contractility from both WT and *Lpar3^−/−^* (Figure 6A and B, Supplementary Table 5). The contractions present throughout the uterus in *Lpar3^+/−^* uteri at GD3 0600 h are of higher velocity (WT = 12.1 μm/s; HET = 41.9 μm/s; KO = 10.0 μm/s), higher amplitude (WT = 175.9 μm; HET = 248.1 μm; and KO = 153.4 μm), and higher wavelength (WT = 658.5; HET = 1759.1 μm; KO = 640.5 μm) compared to both WT and *Lpar3^−/−^* mice. At GD3 1200 h, *Lpar3^+/−^* uteri display higher frequency (WT = 0.0148; HET = 0.0162; KO = 0.0141 Hz) but lower amplitude (WT = 336.0 μm; HET = 182.7 μm; KO = 311.2 μm), lower wavelength (WT = 1723.0 μm; HET = 745.3 μm; KO = 1620.2 μm) and lower velocity (WT = 26.8 μm; HET = 14.3 μm; KO = 22.2 μm/s) contractions compared to WT and *Lpar3^−/−^* uteri. Our data suggest a precocious increase in uterine contractility in *Lpar3^+/−^* uteri compared to both WT and *Lpar3^−/−^* uteri.

While embryo movement patterns for the *Lpar3^−/−^* mice have been studied, and it has been shown that embryos in a *Lpar3^−/−^* uteri implant as clusters [25] resulting in implantation loss; embryo movement in the *Lpar3^+/−^* uteri has not been assessed. Since uterine contractions in the first phase of clustered embryo movement are perturbed in *Lpar3^+/−^* mice (Figure 6), we predicted that these mice would display differential patterns of pre-implantation embryo movement. We observed that at GD3 0900 h in both controls and *Lpar3^+/−^* mice, embryos were approaching the middle of the uterine horn. However, at GD3 1200 h, in *Lpar3^+/−^* mice, embryos started to scatter and separate from each other with 52% of embryos in the oviductal segment, 28% in the middle segment, and 20% in the cervical segment while embryos in the control uteri are still clustered in the middle segment with 61% embryos in the middle, 31% in the oviductal segment, and only 8% in the cervical segment (Supplementary Figure 4). Thus, embryos in the *Lpar3^+/−^* uteri show a similar pattern to the controls initially but at mid-day on GD3, their movement appears faster as seen by distribution in three segments.

### Spatial changes in contractility explain embryo movement trajectories in *Lpar3^−/−^* mice

We observed no global differences between WT and *Lpar3^−/−^* mice when evaluating contractility along the whole uterine horn. However, *Lpar3^−/−^* mice display preferential localization of clustered embryos near the cervix at GD3 1200 h [25, 30, 31]. Even though global contractility appears to be the same, we determined if contractility in *Lpar3^−/−^* uteri is regionally distinct compared to WT mice. Indeed, we observed differences in contractions between WT and *Lpar3^−/−^* uteri when compared in isolated segments of the uterus (oviductal, middle, and cervical segments). At GD3 1200 h, contraction amplitude is reduced in *Lpar3^−/−^* uteri compared to WTs in the oviductal segment (WT = 473.6 μm, KO = 259.4 μm) and the opposite is true in the cervical segment where contraction amplitude is higher in *Lpar3^−/−^* uteri compared to WTs (WT = 212.0 μm, KO = 342.8 μm) (Figure 6C, Supplementary Table 5). At GD3 1200 h, the wavelength of the contractions in the oviductal segment is also reduced in the *Lpar3^−/−^* uteri compared to the WT (WT = 2141.9 μm, KO = 1568.5 μm). These contractility metrics would explain movement of embryo clusters beyond the middle of the uterine horn and closer to the cervix in the *Lpar3^−/−^* uteri compared to controls in the first phase of embryo movement [25].

When evaluating just the WT uteri, we noted that at GD3 0600 h, velocity and wavelength were significantly higher in the middle segment of the uterine horn as compared to the cervical segment, while amplitude and frequency were uniform in all segments (Supplementary Table 6). At GD3 1200 h, amplitude in the oviductal segment was significantly higher than the cervical segment. At this time, both oviductal and middle segments displayed significantly higher values for velocity and wavelength when compared to the cervical segment while frequency was again similar in all segments (Supplementary Table 6). These data suggest that during contraction-dependent embryo movement, the cervical end of the uterus is the least active and the middle of the uterine horn is most contractile. Detection of spatial changes in contractility that correlate with observed embryo movement patterns is an example of how our method can be used to learn new facets of uterine contractility during the pre-implantation stages of early pregnancy.

## Discussion

We describe a spatiotemporal method for quantifying uterine contractions. Our method accounts for contributions from both the longitudinal and circular smooth muscle contractions. Most importantly, ours is the first method that allows quantification for both the cycling uterus contractions and contractions in early pregnancy that are key for pre-implantation embryo movement.

### Contractions during estrous cycle

There are conflicting data on which phase of the estrous cycle is most contractile in the rodent. Some report that diestrus is highly contractile and proestrus is the least contractile [39, 40], and some studies show the exact opposite [20, 41]. Crane and Martin, using laparoscopic video recordings suggested that total contractile activity was lowest at proestrus, increased at estrus, decreased at metestrus, and rose to its highest at diestrus. Using spatiotemporal mapping, Dodds and colleagues [14] clearly indicate that diestrus stage shows maximum contractility. In studying actions of sex hormones on contractions [39], it is found that estrogen is associated with a reduction in contractions due to reduction in calcium channels that are excitatory for contractile activity and increase in K+ channels that are inhibitory for contractile activity. These authors classify contractile activity as either mechanical or electrical. The electrical activity is low in proestrus, increases in estrus and metestrus, and drops in diestrus. However, mechanical activity defined by the propagation of a contraction is highest in diestrus and metestrus. Further, contractile activity can vary by time of day on a certain estrous stage as well [42]. Alternatively, electrical activity–induced contractions in different phases of the mouse estrous cycle suggest that muscle in the estrus stage is most responsive and that in the diestrus stage is least responsive [20]. When force was applied and “spontaneous contractility” under tension transducers was measured [14], contractions in the diestrus phase were quiescent, in the proestrus phase were high-frequency phasic, in the estrus stage were low-frequency phasic, and in the metestrus stage were multivariant. General studies that evoke contractility, either using electrical stimulus or using tension transducers, conclude that diestrus is not contractile; however, when recording spontaneous contractions, we and others [14, 43] observed maximum contractility during the diestrus phase. Our quantitative data agree with the imaging-based studies and shows that diestrus-stage contractility is highly variable, and the uterus is the most contractile in this stage. We predict that applying external force produces different responses and other estrous cycle stages are more responsive to force-induced contractility compared to diestrus. This suggests that induced contractions may indicate the ability of the muscle to respond to stimuli and this may be different from the native spontaneous contractions.

#### Frequency

When comparing numerical values of different waveform metrics, Dodds and colleagues [22] reported frequency at 0 cm water distension to be 1.2 contractions per minute at proestrus, which is comparable to data from Zhang and colleagues [24] that described an overall dominant frequency of ~1 per minute from in vivo recordings. However, the in vivo study also shows that there is a range of frequency from 0.008 to 0.029 Hz. Our proestrus-stage data show contractility with frequency of 0.003–0.029 Hz that aligns well with the range of frequency detected from in vivo studies.

#### Velocity

Dodds and colleagues in their first report suggest contraction velocities from spatiotemporal mapping to be [14] a mean value of 1.3 mm per second (proestrus); 0.9 mm per second (estrus); 1.2 mm per second (estrus); and 0.7 mm per second (diestrus). These values are significantly lower than those reported by Dodds and colleagues in 2021 in the proestrus stage reported as 7.4 mm per second [22]. Our data suggest a maximum median velocity of ~1.4 pixels per second, which translates to 12.8 μm (0.0128 mm) per second in the diestrus stage. Thus, velocity comparisons do not match up across studies. Differences in waveform metrics could be due to the use of different buffers or mouse backgrounds. Our study uses PBS and mixed background mice, while Dodds and colleagues use Krebs buffer and C57Bl6 mice. Thus, comparisons across genotypes or time points with mice on the same background may be more useful and accurate rather than comparing across studies.

#### Amplitude

Maximal strength of the contractile wave, amplitude, has been measured using different methods including applying mechanical tension [14], based on pressure recordings [22], or a single maximal amplitude throughout the uterine horn was calculated using Fast Fourier Transformation (FFT) [24]. We cannot compare our spontaneous contractility data to tension-induced contractility. FFT can only be applied if a single wave propagates throughout the entire uterine horn. Our goal was to calculate local spatial waveform metrics to discern contributions of muscle contractility to embryo movement in the pre-implantation phase, and we did not calculate a maximal amplitude as has been reported in prior methods.

#### Regional differences in contractility

Some studies have reported that caudal uterus (closer to cervix) is more contractile than the rostral uterus (closer to the oviduct) [20]. Stage-specific spatial contractility has also been described where in diestrus, contractility is highest in oviductal segment, while contractility in proestrus is highest in the cervical segment [41]. Dodds and colleagues [14] report that contractions usually originate from the oviductal region in proestrus and estrus and have multiple origin points throughout the horn in metestrus and diestrus. Our area and intensity plots did not support preferential contractile activity in the oviductal or cervical end during proestrus. However, we did observe multiple origin points for contractions throughout the uterine horn in diestrus stage with no directionality bias toward the oviduct or the cervix.

We observed that contractility is highest in progesterone dominant diestrus phase and in the pre-implantation stages. This idea is supported by muscle specific progesterone receptor (PGR) knockout that causes embryo retention in the oviduct and impaired myometrial adaptation to pregnancy [44]. PGR-deficient uteri show reduced response to oxytocin-induced contractility and show reduced expression of calcium homeostasis genes. Transcriptomic analysis suggests that major matrix and muscle genes are regulated by PGR signaling including Myocd and Ccn2. However, to prevent contractions during pregnancy, evolutionarily high progesterone signaling must be linked to uterine quiescence. This apparent discrepancy can be explained by the fact that PGR has two isoforms, PGRA and PGRB. While PGRB promotes relaxation, PGRA promotes contractility in human uterine smooth muscle [45]. Overexpression of PGRB increases gestational length, and overexpression of PGRA increases uterine contractility without affecting gestational length. Transcriptional analysis suggests that PGRB induces muscle relaxation and PGRA is proinflammatory. Thus, depending on which isoform of PGR is expressed and in what tissue, progesterone may induce differential contractile responses as needed for cyclicity and pregnancy success.

### Comparison to other methods for contraction measurement in pregnant state

While there has been a lot of investigation of uterine contractility during different stages of the estrous cycle and during late-term pregnancy, information on contractility during the peri-implantation phase is sparse. Oviductal myogenic contractions have been measured and shown to be responsible for egg/embryo movement during early pregnancy [26]. Using spatiotemporal mapping, oviductal spontaneous contractions were measured although the authors showed that additional contractility can be induced by applying mechanical force. They were simultaneously able to trace the edge of the oocyte and determine that oviductal spontaneous peristaltic like contractions drive movement of the egg. These myogenic contractions and resulting egg movement were inhibited by calcium blockers and were not perturbed when blocking nerve activity. Further while blocking contractility blocks egg and embryo movement, small particle movement (<25 μm) that relies on ciliary activity was not blocked [46]. Oviductal myogenic contractility also relies on interstitial cells of Cajal (ICC). Blocking ICC function by kit antibody or perturbation of ICC networks by bacterial infection both block propagating contractions. There is evidence of ICC in the uterus [14]; however, whether ICC provide pacemakers and initiation of contractility has not been proven.

To conclusively prove that uterine peristaltic spontaneous contractions directly cause uterine embryo movement, simultaneous recordings of embryo movement and uterine wall would be necessary. However, the uterine smooth muscle is ~500 μm thick preventing simultaneous live imaging of the muscle activity and the embryo movement. Thus, in the current study, we are using our previously published patterns for embryo movement [25] and contractility recordings (this study) to evaluate contributions of contractions to embryo movement over time. Our data suggest that early on GD3 of mouse pregnancy, during the first phase of embryo movement, contractile waves are not uniform throughout the horn. With spatial analysis, we determined that the middle of the uterine horn is most contractile while the cervical end of the uterus showed least contractile activity. As the embryos enter the second phase of movement where they scatter and space out, contractions along the entire horn become more rhythmic and uniform. These data would support a more active role for uterine contractions during the first phase of movement as has been observed by use of muscle relaxants in vivo [25].

### Comparison of image analysis methods

Prior studies that measure contractions based on video-recording generate kymographs or spatiotemporal maps that were initially developed for the intestine [47] and then applied to the oviduct [26] and the non-pregnant uterus [14, 22]. These methodologies produce very similar data to our area-based plots. Zhang and colleagues [24] used eight different equally spaced locations along the uterine horn to assess the movement of the uterine horn edges away from a central line through the uterine horn and then applied an area-based calculation. Our method utilizes changes along the length of the uterine horn and accounts for changes in area, but we also evaluate changes in contractility along the uterine horn using tomato intensity. These changes in intensity are particularly useful to study spatial distribution of contractions along the uterus in pre-implantation stages of pregnancy.

Below, we summarize the advantages and limitations of our method.

#### Advantages

Our method presents the following advantages over previously published methods:

(i) We can record any length of uterine horn, and thus, we are able to record contractions from both CD1 (Figures 4 and 5) and C57Bl6 mouse background (Figure 6). The limit for uterine horn length is determined by the objective lens used for capturing the tomato+ images.
(ii) We are able to image diestrus and early pregnancy stages that have stronger and more variable contractions.
(iii) Using intensity plots for measuring contractions, we have the spatial resolution to separate effects on different segments of the uterus—closer to the oviduct, middle of the uterine horn, and closer to the cervix.
(iv) Since our recordings are ex vivo and not in vivo, in our datasets, contractility is not influenced by an anesthetic.
(v) Despite being an ex vivo method, our initial measurements for frequency of contractions match up with the in vivo data [24].
(vi) Our method is also amenable to short-term manipulation in the culture dish with fast-acting small-molecule inhibitors and activators. Alternatively, mice can be treated in vivo for longer-acting molecules such as ovarian hormones and still be assessed later.

#### Limitations

Although our method presents an advance and allows recording of pre-implantation-stage uteri, there is room for improvement of the method. The following limitations and possible resolutions are noted:

(i) Although the area plots can be obtained from any mouse line, a tomato allele needs to be bred with the mice under investigation in order to collect intensity data. To avoid breeding the tomato expressing transgene, transient embryo transfection could be performed within the uterus with an adenovirus carrying the reporter gene [48].
(ii) Animals need to be sacrificed; thus, the same animal cannot be imaged repeatedly for different stages. Conducting imaging in vivo would be an alternative; however, current methodologies that allow in vivo imaging are terminal and the same mouse cannot be imaged through multiple estrous stages.
(iii) Pinning the uterine horn in its in vivo configuration is a manual component of the method. Stretching the uterus too much will prevent in vivo-like measurements, and leaving slack in the uterus may prevent the computational workflow from being applied to the captured images. Again, conducting the imaging in vivo in the native configuration is the best alternative.
(iv) Our recordings are done promptly after the animal is sacrificed, and we record contractility for 5 min. Any waves that have a frequency larger than 5 min will be missed. Imaging and quantifying waves for longer than 5 min will allow assessment of longer waves ex vivo; however, how closely these waves reflect in vivo contractility would need to be determined.
(v) The buffer used while imaging can impact contractility, and this should be kept in mind when comparing data generated from our method to results from other studies.
(vi) The intensity-based map is derived from the mean intensity of each ring segment. The intensity of the images within some segments exhibits considerable heterogeneity in the vertical direction. Incorporating the full-intensity distribution along the vertical direction would indeed provide a more comprehensive analysis. This limitation will be addressed in future investigations as we extend our analysis from a 3D to a four-dimensional problem space. This will improve the capabilities of the method.
(vii) Our method allows for relative quantification of two stages, and absolute values of waveform metrics should be used cautiously. Our method was used to measure contractions across different mouse backgrounds, and we did observe that at pregnancy stage GD3 1200 h, contractility metrics for CD1 mice (Figure 5) differed from WT litter mates on C57Bl6 background (Figure 6). These data suggest variations in contractility due to genetic backgrounds and further emphasize that these methods should be used to compare tissues on the same genetic background.
(viii) Although we obtain intensity plots and much additional data, we are still limited by manual calling of waves and calculating metrics based on those calls. Further, many smaller waves can shadow the larger waves in the intensity plots making it harder to analyze larger waves in a highly contractile uterus. For both these reasons, the method of calling waves needs to be automated and is a subject of future investigations. Image analysis tools like Kymobutler [49] can help with automating the process of tracking the waves on the spatiotemporal plots; however, the output will need to be integrated for waveform metric calculation and is a subject of our future work.

### LPAR3: a stimulant or relaxant for uterine contractions?

The LPA-LPAR3 signaling pathway has been implicated in embryo movement and uterine contractility [25, 28, 29]. *Lpar3^−/−^* uteri display embryo clusters that move past the middle of the uterine horn in the first phase of movement and a failure of embryo clusters to separate in the second phase of embryo movement. In vitro mechanical transducer–induced assays suggested that while control uteri can respond to LPA-LPAR3 agonist, *Lpar3^−/−^* uteri fail to respond to the agonist as measured by contractility, however, these uteri contract in the presence of acetyl choline [28]. Further, LPA can dose-dependently increase contraction amplitude in estrus stage rat uteri in in vitro uterine strips and in vivo [50, 51]. Additionally, a study with gilts (pig uteri) suggested that LPA signaling enhances contractility in early pregnancy uteri but not non-pregnant uteri [52]. All of these studies suggest that LPA-LPAR3 pathway activation is associated with an increase in contractions. However, movement of embryo clusters past the middle of the uterine horn and closer to the cervix in the contraction-dependent phase of embryo movement would predict that spontaneous contractions are increased in LPAR3-deficient uteri. Our method demonstrates a spatial disruption in *Lpar3^−/−^* uterine contractions. While in WT uteri, contractions are active in the oviductal segment of the uterine horn, contractions in *Lpar3^−/−^* uteri are higher in the cervical segment of the uterine horn. Further, *Lpar3^+/−^* uteri also display higher contractility that correlates with faster movement of embryos in these uteri. It is critical to note that while the increase in contractility was observed at GD3 0600 h (Figure 6), the faster embryo movements were observed a little later at GD3 1200 h (Supplementary Figure 4). These data suggest that there are other factors in addition to contractility that may fine-tune embryo movement patterns. Thus, loss of LPA-LPAR3 activity may be related to enhanced contractility in a regional manner in the first phase of embryo movement. LPA agonists and antagonists can affect contractility differently in pregnant uteri when compared to cycling uteri [52]; thus, there may be a hormonal component in regulating when LPA signaling activates contractions or inhibits contractions.

### Future directions

Contractility, especially in the context of object movement, is complex and has many facets, including what kind of stimuli is used to induce contractility and what the hormonal milieu is. The least studied in the context of uterine contractions is whether the diameter of the uterine lumen impacts propagation of uterine contractions. Proestrus and estrus-staged uteri are fluid-filled due to estrogen-regulated luminal epithelium secretions [53]. On the other hand, diestrus-staged uteri show narrower luminal openings [53] that likely support contractile waves to travel along greater distances. Thus, hormonal impacts cannot be considered independent of the effects of ovarian hormones on the luminal epithelium structure and volume and nature of luminal epithelial secretions. Theoretically, estrogen in the proestrus and estrus stages may show reduced propagation of contractile waves due to an open lumen; however, supplementing with estrogen during progesterone-dominant pre-implantation may increase contractility due to a closed lumen. This idea is supported by our observation that embryos travel farther along the uterine horn when treated with estrogen during pre-implantation stages [54]. Progesterone may permit luminal closure but by itself may have an inhibitory effect on how far an individual contraction travels. Thus, several open questions remain. How do absolute levels of ovarian hormones or an estrogen:progesterone ratio regulate uterine lumen structure, fluid accumulation in the lumen and movement of objects through the lumen? Do objects such as eggs and embryos in the uterine horn act as pacemakers to trigger spatially distinct contractile waves? Our method can now allow researchers to start addressing some of these scenarios. In the long term, understanding how contractility regulates early embryo movement and how factors such as ovarian hormones or signaling molecules such as LPA regulate uterine contractions can be useful in modulating contractility during embryo transfer to improve outcomes for in vitro fertilization and artificial reproductive technologies in the clinic.

## Supplementary Material

Supplementary_Figure_1_ioae071

Supplementary_Figure_2_ioae071

Supplementary_Figure_3_ioae071

Supplementary_Figure_4_ioae071

Supplementary_Tables_ioae071

Supplementary_Methods_ioae071

Supplementary_Video_1_ioae071

Supplementary_Video_2_ioae071

Supplementary_Video_3_ioae071

Supplementary_Video_4_ioae071

Legends_for_supplementary_videos_ioae071

## Data Availability

Data generated in the manuscript are available upon reasonable request to the corresponding author.
