# Supplementary figures and images for: Imaging the dynamics of murine uterine contractions in early pregnancy
Source: Biol Reprod. 2024 May 7;110(6):1175–90. doi: 10.1093/biolre/ioae071 (PMC11180618; doi:10.1093/biolre/ioae071)

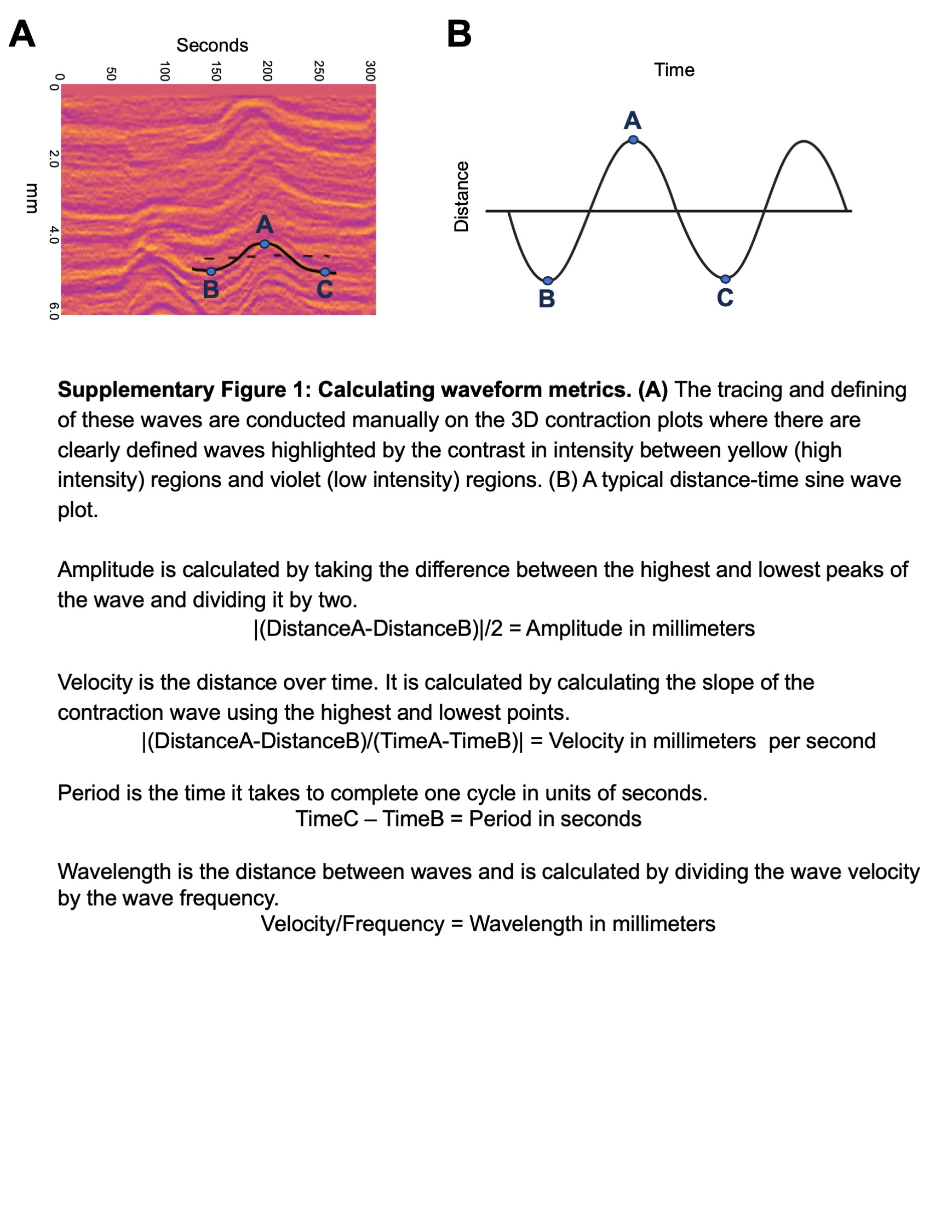

Supplement: Supplementary_Figure_1_ioae071 [file supplementary_figure_1_ioae071.jpeg]

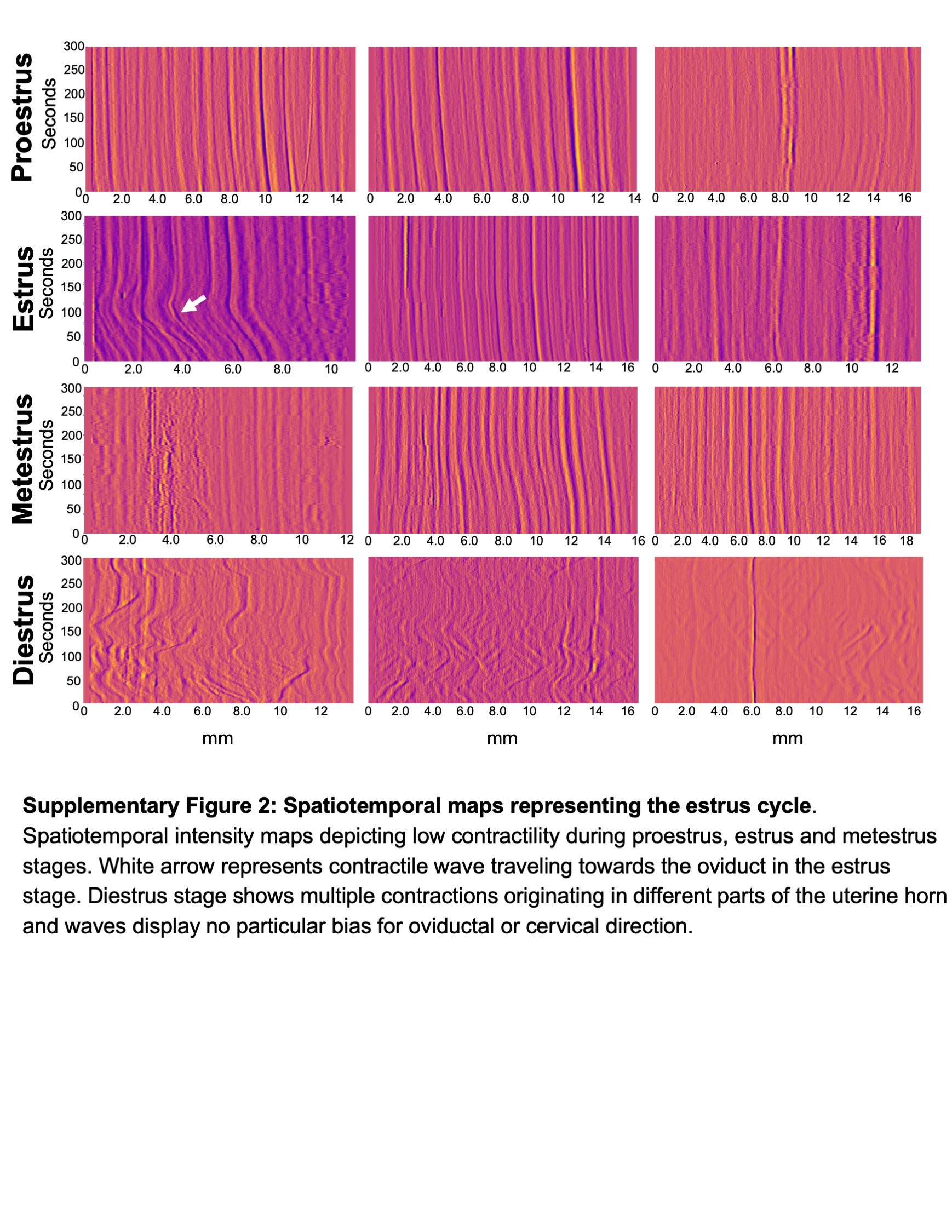

Supplement: Supplementary_Figure_2_ioae071 [file supplementary_figure_2_ioae071.jpeg]

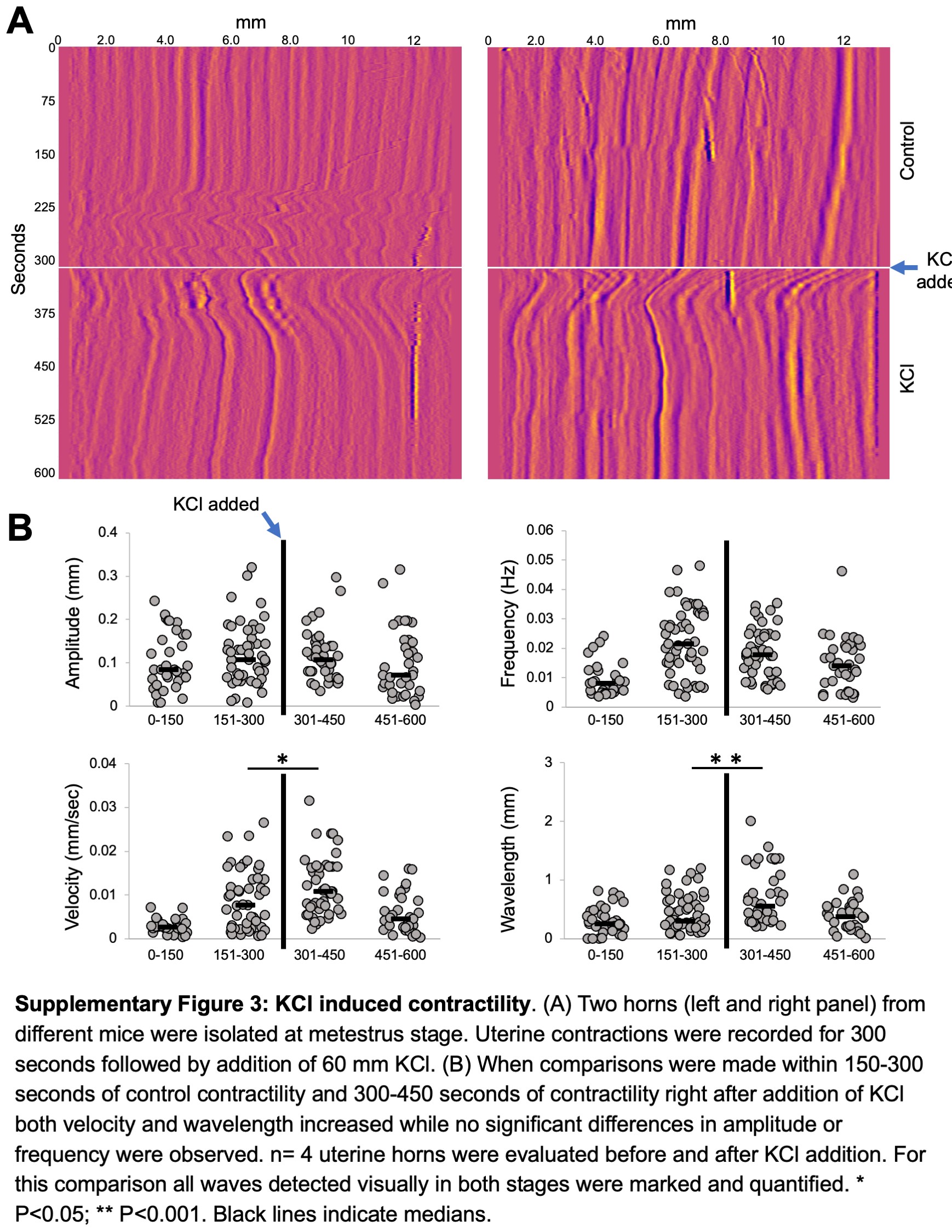

Supplement: Supplementary_Figure_3_ioae071 [file supplementary_figure_3_ioae071.jpeg]

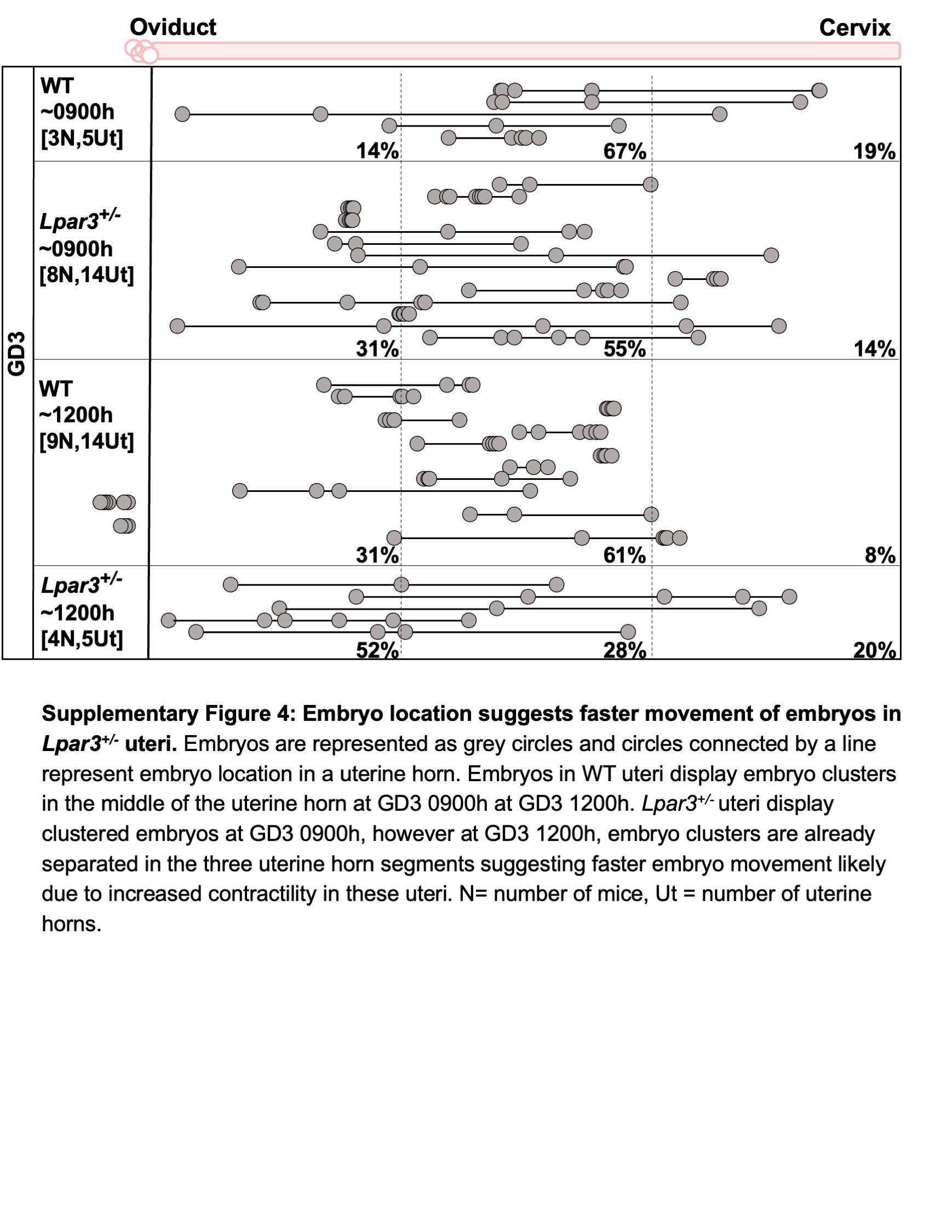

Supplement: Supplementary_Figure_4_ioae071 [file supplementary_figure_4_ioae071.jpeg]
