# Supplementary material for: Imaging the dynamics of murine uterine contractions in early pregnancy
Source: Biol Reprod. 2024 May 7;110(6):1175–90. doi: 10.1093/biolre/ioae071 (PMC11180618; doi:10.1093/biolre/ioae071)
Supplement: Supplementary_Tables_ioae071 [file supplementary_tables_ioae071.docx]

**Supplementary Table 1:** Median values of waveform metrics from 3D intensity plots or 3D area plots and statistical differences between calculated metrics. (Stats for Figure 4)

**Proestrus**

|  | Units | Intensity | Area | Significance |
| --- | --- | --- | --- | --- |
| Amplitude | μm | 128.6 | 108.3 | NS |
| Velocity | μm per second | 4.01 | 2.12 | p<0.05 |
| Frequency | hz | 0.006 | 0.008 | NS |
| Wavelength | μm | 572.8 | 550.3 | NS |

NS: not significant

**Diestrus**

|  | Units | Intensity | Area | Significance |
| --- | --- | --- | --- | --- |
| Amplitude | μm | 263.9 | 311.2 | NS |
| Velocity | μm per second | 26.3 | 34.8 | NS |
| Frequency | hz | 0.015 | 0.017 | p<0.05 |
| Wavelength | μm | 1488.5 | 1484.0 | NS |

NS: not significant

**Supplementary Table 2:** Median values of waveform metrics from 3D intensity plots for different phases of estrus cycles followed by pairwise comparisons for statistical differences between calculated metrics. (Stats for Figure 5)

**Median Values**

|  | Units | Proestrus | Estrus | Metestrus | Diestrus |
| --- | --- | --- | --- | --- | --- |
| Amplitude | mm | 110.5 | 83.4 | 166.9 | 189.4 |
| Velocity | mm per second | 3.6 | 2.0 | 5.7 | 12.8 |
| Frequency | hertz | 0.005 | 0.006 | 0.007 | 0.016 |
| Wavelength | mm | 577.4 | 419.5 | 766.8 | 802.9 |

**Pairwise statistical comparisons**

| Amplitude | Proestrus | Estrus | Metestrus | Diestrus |
| --- | --- | --- | --- | --- |
| Proestrus | - | NS | p<0.001 | p<0.0001 |
| Estrus | - | - | p<0.0001 | p<0.0001 |
| Metestrus | - | - | - | p<0.05 |
| Diestrus | - | - | - | - |
|  |  |  |  |  |
| Velocity | Proestrus | Estrus | Metestrus | Diestrus |
| Proestrus | - | p<0.001 | p<0.05 | p<0.0001 |
| Estrus | - | - | p<0.0001 | p<0.0001 |
| Metestrus | - | - | - | p<0.0001 |
| Diestrus | - | - | - | - |
|  |  |  |  |  |
| Frequency | Proestrus | Estrus | Metestrus | Diestrus |
| Proestrus | - | NS | p<0.05 | p<0.0001 |
| Estrus | - | - | p<0.05 | p<0.0001 |
| Metestrus | - | - | - | p<0.0001 |
| Diestrus | - | - | - | - |
|  |  |  |  |  |
| Wavelength | Proestrus | Estrus | Metestrus | Diestrus |
| Proestrus | - | p<0.05 | p<0.05 | p<0.0001 |
| Estrus | - | - | p<0.0001 | p<0.0001 |
| Metestrus | - | - | - | p<0.0001 |
| Diestrus | - | - | - | - |

NS: not significant

**Supplementary Table 3:** Median values of waveform metrics from 3D intensity plots for metestrus stage in wildtype with the addition of a KCl dose followed by pairwise comparisons for statistical differences between calculated metrics. (Stats for Supplementary Figure 2)

**Median Values**

|  |  | Control | | KCl | |
| --- | --- | --- | --- | --- | --- |
|  | Units | 0-150 secs | 151-300 secs | 301-450 secs | 451-600 secs |
| Amplitude | μm | 85.7 | 108.3 | 108.3 | 72.2 |
| Velocity | μm per second | 2.6 | 7.7 | 10.9 | 4.6 |
| Frequency | hertz | 0.008 | 0.022 | 0.018 | 0.014 |
| Wavelength | μm | 261.6 | 306.7 | 559.3 | 378.9 |

**Pairwise statistical comparisons**

| Amplitude | Control Early | Control Late | KCl Early | KCl Late |
| --- | --- | --- | --- | --- |
| Control Early | - | NS | NS | NS |
| Control Late | - | - | NS | NS |
| KCl Early | - | - | - | NS |
| KCl Late | - | - | - | - |
|  |  |  |  |  |
| Frequency | Control Early | Control Late | KCl Early | KCl Late |
| Control Early | - | p<0.0001 | p<0.0001 | p<0.05 |
| Control Late | - | - | NS | p<0.001 |
| KCl Early | - | - | - | p<0.05 |
| KCl Late | - | - | - | - |
|  |  |  |  |  |
| Velocity | Control Early | Control Late | KCl Early | KCl Late |
| Control Early | - | p<0.0001 | p<0.0001 | p<0.05 |
| Control Late | - | - | p<0.05 | p<0.05 |
| KCl Early | - | - | - | p<0.0001 |
| KCl Late | - | - | - | - |
|  |  |  |  |  |
| Wavelength | Control Early | Control Late | KCl Early | KCl Late |
| Control Early | - | NS | p<0.0001 | NS |
| Control Late | - | - | p<0.001 | NS |
| KCl Early | - | - | - | p<0.05 |
| KCl Late | - | - | - | - |

NS: not significant

**Supplementary Table 4:** Median values of waveform metrics from 3D intensity plots for pre-implantation pregnancy time points followed by pairwise comparisons for statistical differences between calculated metrics. (Stats for Figure 5)

**Median Values**

|  | Units | GD3 0600h | GD3 1200h | GD3 1800h | GD4 1200h | Diestrus |
| --- | --- | --- | --- | --- | --- | --- |
| Amplitude | μm | 171.4 | 81.2 | 142.1 | 194.0 | 189.4 |
| Velocity | μm per second | 8.9 | 11.1 | 5.9 | 9.4 | 12.8 |
| Frequency | hertz | 0.013 | 0.012 | 0.011 | 0.010 | 0.016 |
| Wavelength | μm | 631.5 | 487.1 | 545.8 | 929.2 | 802.9 |

**Pairwise comparisons**

| Amplitude | GD3 0600h | GD3 1200h | GD3 1800h | GD4 1200h | Diestrus |
| --- | --- | --- | --- | --- | --- |
| GD3 0600h | - | p<0.0001 | NS | NS | p<0.05 |
| GD3 1200h | - | - | p<0.05 | p<0.05 | p<0.0001 |
| GD3 1800h | - | - | - | NS | p<0.0001 |
| GD4 1200h | - | - | - | - | p<0.05 |
| Diestrus | - | - | - | - | - |
|  |  |  |  |  |  |
| Velocity | GD3 0600h | GD3 1200h | GD3 1800h | GD4 1200h | Diestrus |
| GD3 0600h | - | NS | NS | NS | p<0.0001 |
| GD3 1200h | - | - | p<0.001 | NS | p<0.0001 |
| GD3 1800h | - | - | - | p<0.05 | p<0.0001 |
| GD4 1200h | - | - | - | - | p<0.0001 |
| Diestrus | - | - | - | - | - |
|  |  |  |  |  |  |
| Frequency | GD3 0600h | GD3 1200h | GD3 1800h | GD4 1200h | Diestrus |
| GD3 0600h | - | NS | NS | NS | p<0.05 |
| GD3 1200h | - | - | NS | p<0.05 | NS |
| GD3 1800h | - | - | - | NS | p<0.05 |
| GD4 1200h | - | - | - | - | p<0.0001 |
| Diestrus | - | - | - | - | - |
|  |  |  |  |  |  |
| Wavelength | GD3 0600h | GD3 1200h | GD3 1800h | GD4 1200h | Diestrus |
| GD3 0600h | - | NS | NS | NS | p<0.0001 |
| GD3 1200h | - | - | NS | p<0.05 | p<0.0001 |
| GD3 1800h | - | - | - | p<0.05 | p<0.0001 |
| GD4 1200h | - | - | - | - | p<0.05 |
| Diestrus | - | - | - | - | - |

NS: not significant

**Supplementary Table 5:** Median values of waveform metrics from 3D intensity plots for pre-implantation pregnancy time points in wildtype, *Lpar3^+/-^* and *Lpar3^-/-^* uteri followed by pairwise comparisons for statistical differences between calculated metrics. (Stats for Figure 6)

Median Values

|  |  | **GD3 0600h** | | | **GD3 1200h** | | |
| --- | --- | --- | --- | --- | --- | --- | --- |
|  | Units | Wildtype | *Lpar3^+/-^* | *Lpar3^-/-^* | Wildtype | *Lpar3^+/-^* | *Lpar3^-/-^* |
| Amplitude | μm | 175.9 | 248.1 | 153.4 | 336.0 | 182.7 | 311.2 |
| Frequency | hertz | 0.017 | 0.012 | 0.017 | 0.015 | 0.016 | 0.014 |
| Velocity | μm per second | 12.1 | 41.9 | 10.0 | 26.8 | 14.3 | 22.2 |
| Wavelength | μm | 658.5 | 1759.1 | 640.5 | 1723.0 | 745.3 | 1620.2 |
| Period | seconds | 59.6 | 66.8 | 59.4 | 67.6 | 61.2 | 70.7 |

Pairwise statistical comparisons

| **GD3 0600h** | | | |
| --- | --- | --- | --- |
| Amplitude | WT | *Lpar3^+/-^* | *Lpar3^-/-^* |
| Wildtype | - | p<0.001 | NS |
| *Lpar3^+/-^* | - | - | p<0.0001 |
| *Lpar3^-/-^* | - | - | - |
|  |  |  |  |
| Velocity | WT | *Lpar3^+/-^* | *Lpar3^-/-^* |
| Wildtype | - | p<0.0001 | NS |
| *Lpar3^+/-^* | - | - | p<0.0001 |
| *Lpar3^-/-^* | - | - | - |
|  |  |  |  |
| Frequency | WT | *Lpar3^+/-^* | *Lpar3^-/-^* |
| Wildtype | - | p<0.001 | NS |
| *Lpar3^+/-^* | - | - | p<0.0001 |
| *Lpar3^-/-^* | - | - | - |
|  |  |  |  |
| Wavelength | WT | *Lpar3^+/-^* | *Lpar3^-/-^* |
| Wildtype | - | p<0.001 | NS |
| *Lpar3^+/-^* | - | - | p<0.0001 |
| *Lpar3^-/-^* | - | - | - |

| **GD3 1200h** | | | |
| --- | --- | --- | --- |
| Amplitude | WT | *Lpar3^+/-^* | *Lpar3^-/-^* |
| Wildtype | - | p<0.0001 | NS |
| *Lpar3^+/-^* | - | - | p<0.0001 |
| *Lpar3^-/-^* | - | - | - |
|  |  |  |  |
| Velocity | WT | *Lpar3^+/-^* | *Lpar3^-/-^* |
| Wildtype | - | p<0.0001 | NS |
| *Lpar3^+/-^* | - | - | p<0.05 |
| *Lpar3^-/-^* | - | - | - |
|  |  |  |  |
| Frequency | WT | *Lpar3^+/-^* | *Lpar3^-/-^* |
| Wildtype | - | p<0.05 | NS |
| *Lpar3^+/-^* | - | - | p<0.05 |
| *Lpar3^-/-^* | - | - | - |
|  |  |  |  |
| Wavelength | WT | *Lpar3^+/-^* | *Lpar3^-/-^* |
| Wildtype | - | p<0.0001 | NS |
| *Lpar3^+/-^* | - | - | p<0.0001 |
| *Lpar3^-/-^* | - | - | - |

NS: not significant

Segment-wise statistical comparison (for significant differences only)

| **GD3 1200h** | Amplitude (μm) | |  |
| --- | --- | --- | --- |
|  | Wildtype | *Lpar3^-/-^* | Significance |
| Oviduct | 473.6 | 259.4 | p<0.05 |
| Cervix | 212.0 | 342.8 | p<0.05 |

| **GD3 1200h** | Wavelength (μm) | |  |
| --- | --- | --- | --- |
|  | Wildtype | *Lpar3^-/-^* | Significance |
| Oviduct | 2141.9 | 1568.5 | p<0.05 |

**Supplementary Table 6:** Median values of waveform metrics from 3D intensity plots for pre-implantation pregnancy time points in wildtype but split in different segments of the uterine horn.

**Median Values**

|  | Units | GD3 0600h | GD3 1200h | P value |
| --- | --- | --- | --- | --- |
| Amplitude |  |  |  |  |
| Oviduct | μm | 148.8 | 473.6 | p<0.0001 |
| Middle | μm | 194.0 | 333.8 | p<0.05 |
| Cervix | μm | 144.3 | 212.0 | NS |
|  |  |  |  |  |
| Frequency |  |  |  |  |
| Oviduct | hertz | 0.017 | 0.014 | p<0.05 |
| Middle | hertz | 0.016 | 0.015 | NS |
| Cervix | hertz | 0.017 | 0.016 | NS |
|  |  |  |  |  |
| Velocity |  |  |  |  |
| Oviduct | μm per second | 10.6 | 27.5 | p<0.001 |
| Middle | μm per second | 16.1 | 32.1 | p<0.0001 |
| Cervix | μm per second | 8.8 | 20.3 | p<0.05 |
|  |  |  |  |  |
| Wavelength |  |  |  |  |
| Oviduct | μm | 631.5 | 2141.9 | p<0.0001 |
| Middle | μm | 965.3 | 2383.1 | p<0.0001 |
| Cervix | μm | 424.0 | 1203.0 | p<0.001 |

**Pair-wise statistical comparisons**

| **GD3 0600h** | | | |
| --- | --- | --- | --- |
| **Amplitude** | Oviduct | Middle | Cervix |
| Oviduct | - | NS | NS |
| Middle | - | - | NS |
| Cervix | - | - | - |
| **Frequency** |  |  |  |
| Oviduct | - | NS | NS |
| Middle | - | - | NS |
| Cervix | - | - | - |
| **Velocity** |  |  |  |
| Oviduct | - | NS | NS |
| Middle | - | - | p<0.05 |
| Cervix | - | - | - |
| **Wavelength** |  |  |  |
| Oviduct | - | NS | NS |
| Middle | - | - | p<0.05 |
| Cervix | - | - | - |

NS: not significant

| **GD3 1200h** | | | |
| --- | --- | --- | --- |
| **Amplitude** | Oviduct | Middle | Cervix |
| Oviduct | - | NS | p<0.05 |
| Middle | - | - | NS |
| Cervix | - | - | - |
| **Frequency** |  |  |  |
| Oviduct | - | NS | NS |
| Middle | - | - | NS |
| Cervix | - | - | - |
| **Velocity** |  |  |  |
| Oviduct | - | NS | p<0.05 |
| Middle | - | - | p<0.05 |
| Cervix | - | - | - |
| **Wavelength** |  |  |  |
| Oviduct | - | NS | p<0.05 |
| Middle | - | - | p<0.05 |
| Cervix | - | - | - |

NS: not significant
