# Supplementary material for: Imaging the dynamics of murine uterine contractions in early pregnancy
Source: Biol Reprod. 2024 May 7;110(6):1175–90. doi: 10.1093/biolre/ioae071 (PMC11180618; doi:10.1093/biolre/ioae071)
Supplement: Supplementary_Methods_ioae071 [file supplementary_methods_ioae071.docx]

----------------------------------------- Summary of the method with images --------------

STEPS

<https://docs.opencv.org/4.x/d4/d86/group__imgproc__filter.html#gaabe8c836e97159a9193fb0b11ac52cf1>

-- Smooth with Gaussian in time

cv2.GaussianBlur ( COLUMN, 3, 3)

-- Smooth with Gaussian in Space

cv2.GaussianBlur ( COLUMN, 5, 5)


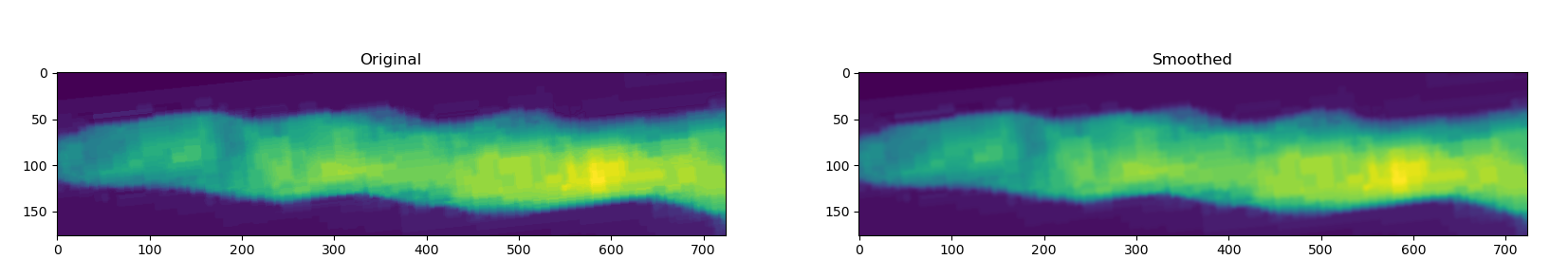


--- Compute Sobel from Smooth video

<https://docs.opencv.org/3.4/d2/d2c/tutorial_sobel_derivatives.html>

Sobelx = cv2.Sobel( img, 0, 1, kernel_size = 5)

Soboely = cv2.Sobel( img, 1, 0, kernel_size = 5)

Sobel Output = 2*sobelx + sobely


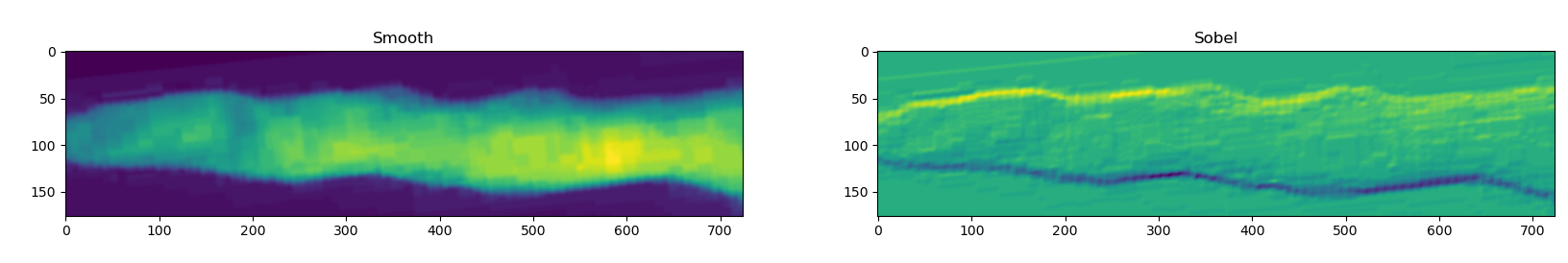


-- Detect top position from the cumulative sum of the Sobel and a predefined threshold. Manually tuned for each video with a default value of **1600**


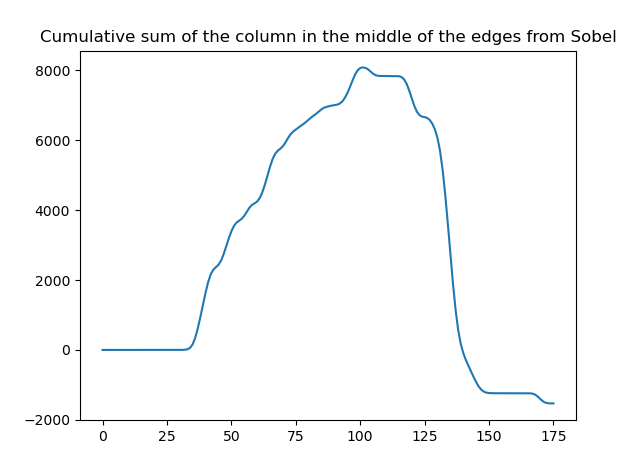


-- Detect bottom position from the ‘flipped’ cumulative distribution, manually adjusted with a default threshold of 1900.


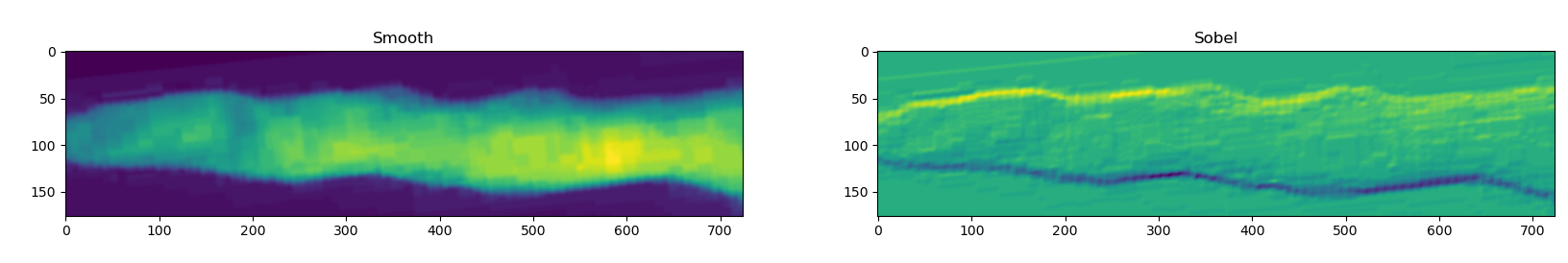


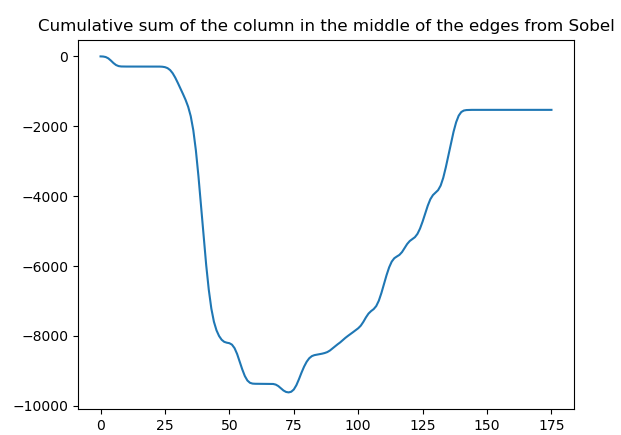


Output


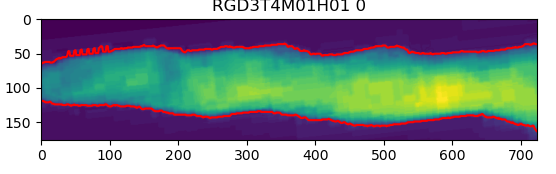


-- Apply median filter to the locations. With default kernel size of 5

scipy.signal

medfilt(top_pos[cur_frame], median_filt_size_top)


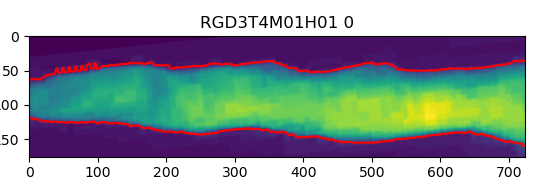


-- CubicSplines

Interpolates the curves using cubic interpolation. The number of points is around **cols/1000 * 50** for the top and **cols/1000*100** at the bottom. Around something like 36 and 70 in the example below.


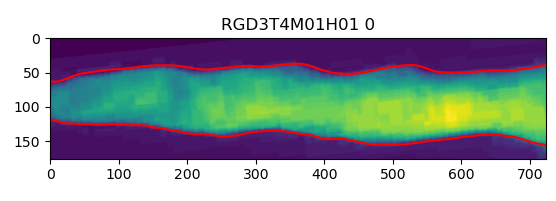


-- Saves mean intensities, bottom, top, and area (bottom - pos)

======================== Second file ==========================

For each previous sequence (area, top, bottom, etc) computes:

1. (name should be space variations) Vertical edges **(sobel 0,1 ksize=21)**→ This is used to detect space variations (by column) independent of the areas ‘far away’ from the current location.
2. (name should be time variations) Horizontal edges (**sobel 1,0 ksize =31)** → This is used to detect changes between time (not very clear most of the time)
3. Filtered image
   1. Compute low frequencies from a running mean of 40 columns
   2. Removes the low frequencies from the original
   3. Smooths a curve with a running mean of 3 columns


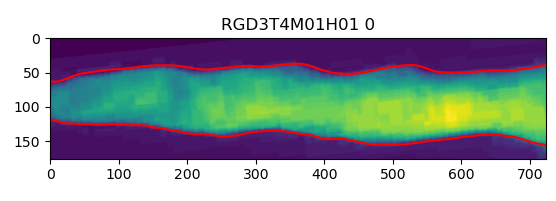


Area


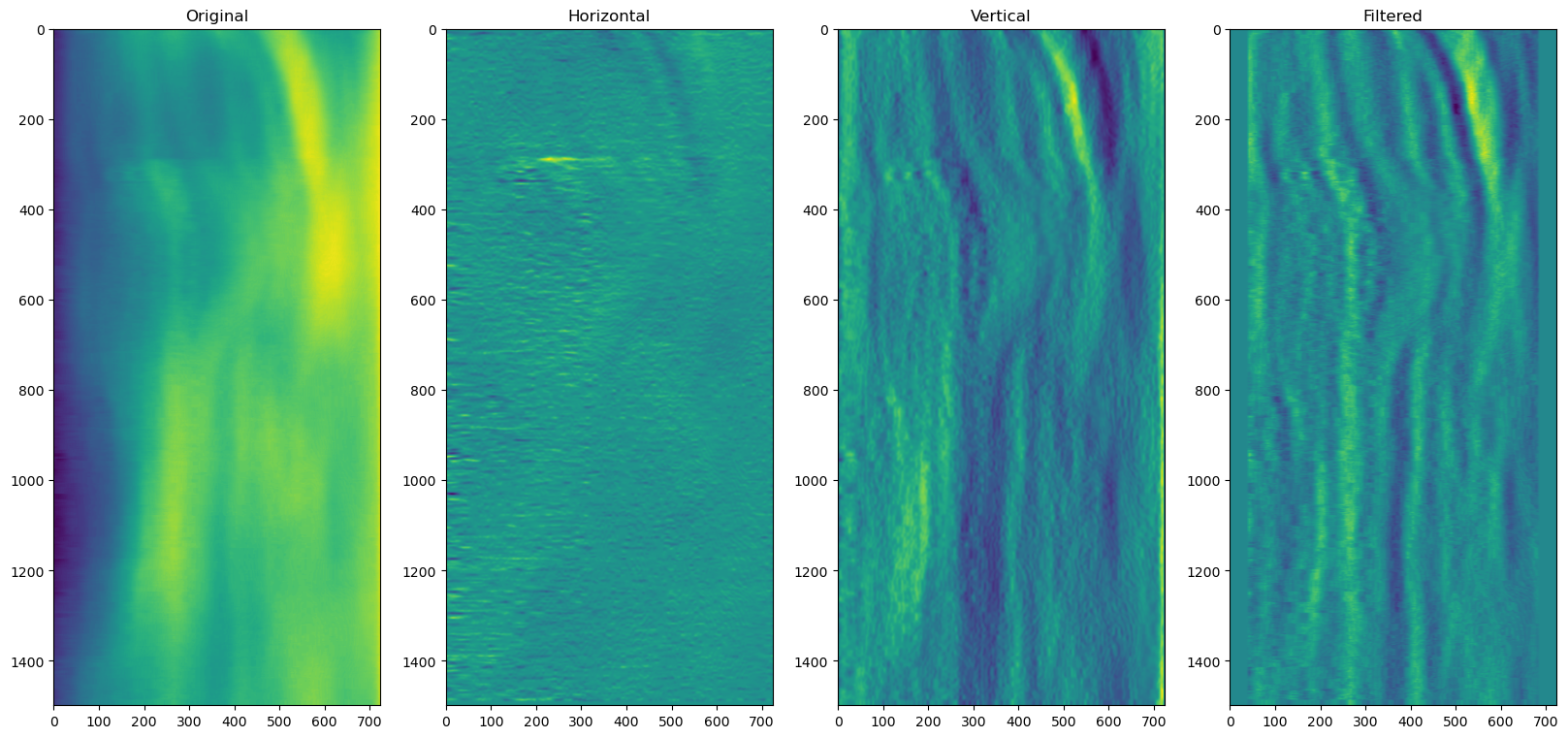


Intensity → Number of cells


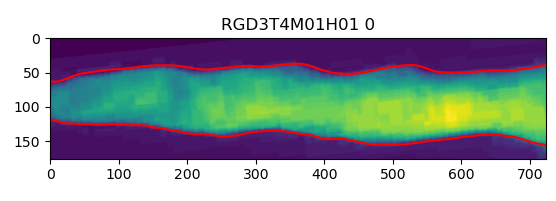


Mean intensities


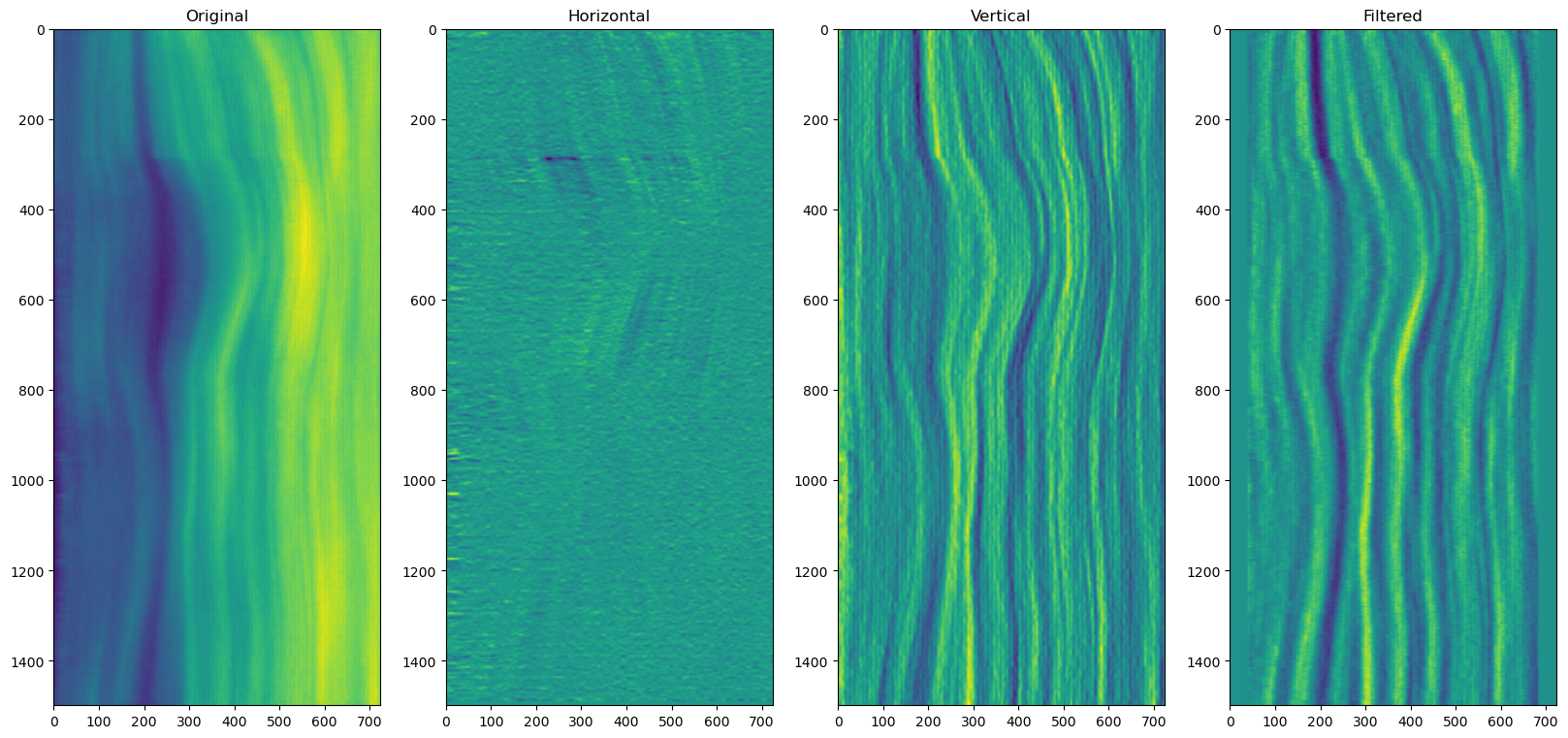


Bottom


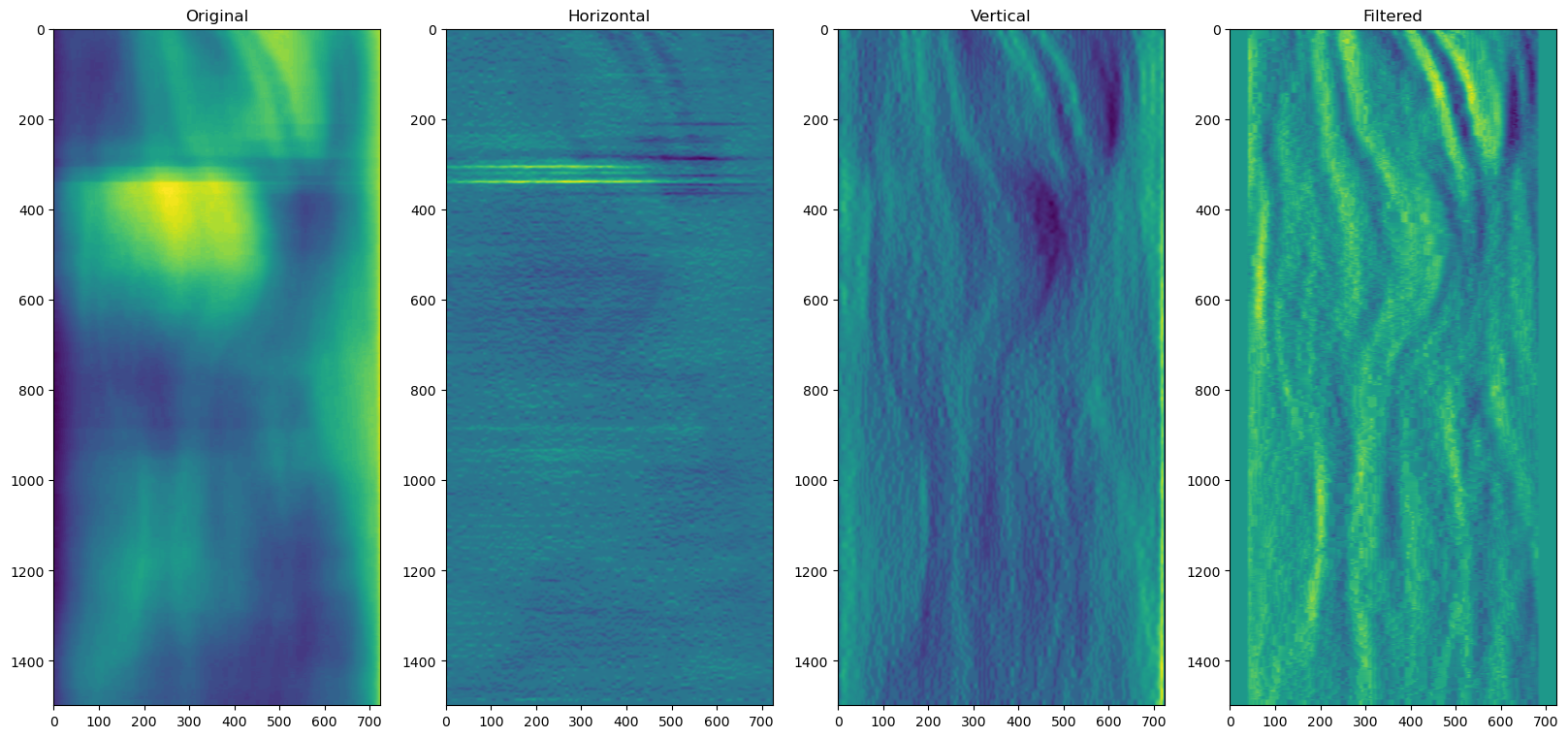


======================== Data Output==========================

A Mask Area Folder is created after running step 1 of the code. In this folder the mask area graphs have images with marked changes in intensity (blue (low intensity)/green (medium intensity)/yellow (high intensity)) and uterine horn borders (in red) from each frame of the .AVI video. These mask area files are used for constructing the area and mean intensity graphs in step 2 of the code.


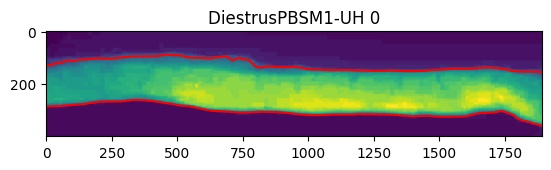


Area Graphs (jpg) and Mean intensity graphs (jpg)

Normalized graphs in a jpg format are generated. Note that the area or intensity changes as depicted by the color are represented in normalized units. Wave coordinates cannot be extracted from these images so .html files need to be used (see next point).


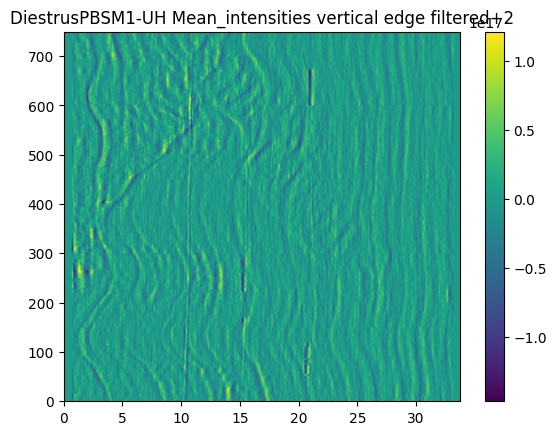

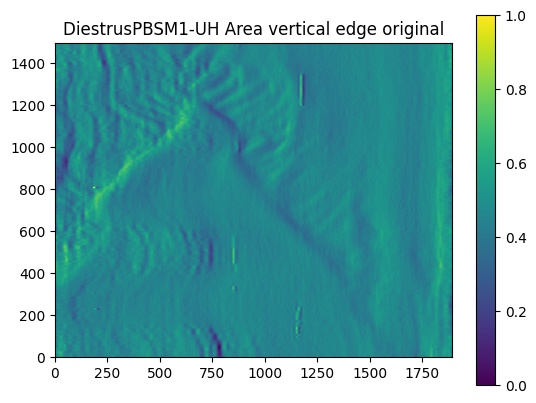


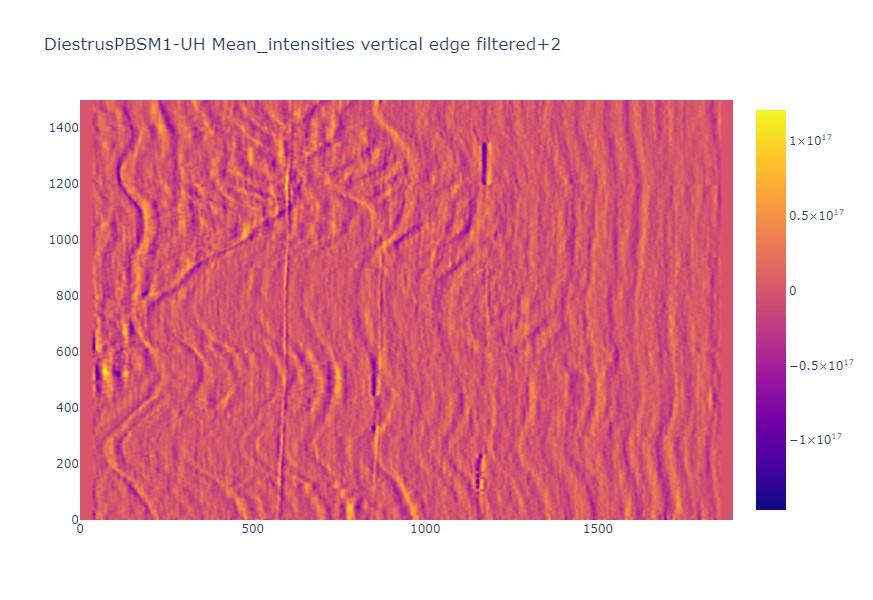

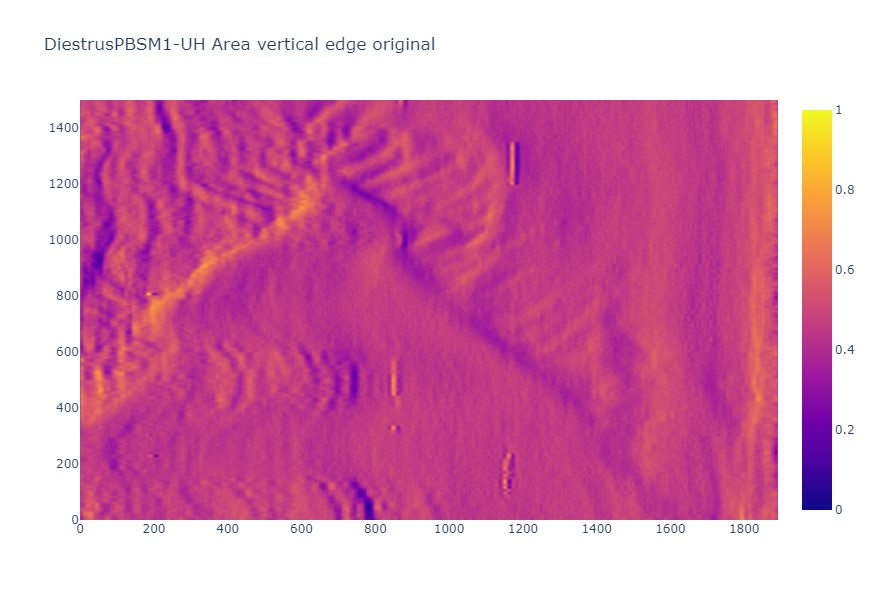
Area graphs or Mean Intensity Graphs (html): this output html file can be opened in a browser and wave coordinates are manually extracted by marking waves and calculating wave form metrics.

**Tracing and Quantifying Waveform Metrics**


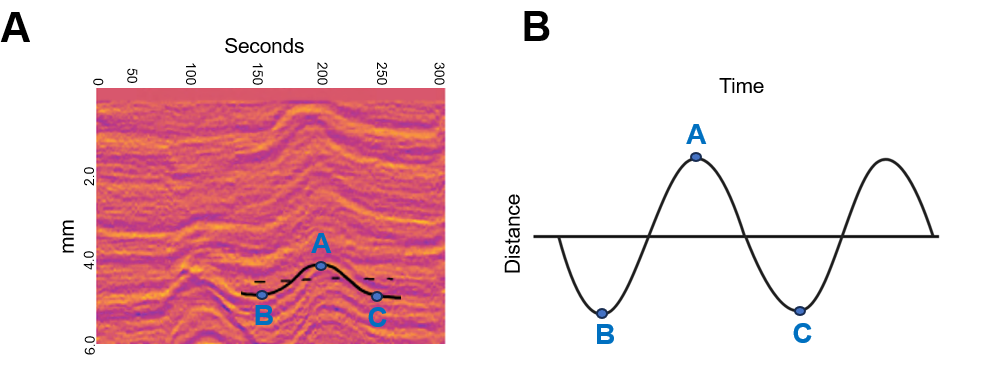


The tracing and defining of these waves are conducted manually on the 3D contraction plots where there are clearly defined waves highlighted by the contrast in intensity between yellow (high intensity) regions and violet (low intensity) regions. Example calculations are conducted below using the example wave marked above. Calculation points are marked and labelled. [A = (190 sec, 4.3 mm), B = (150 sec, 5.2 mm), C = (247 sec, 5.2 mm)]

Amplitude is calculated by taking the difference between the highest and lowest peaks of the wave and dividing it by two.

|(DistanceA-DistanceB)|/2 = Amplitude in millimeters  (1)

|4.3-5.2|/2 = 0.45 mm (Amplitude)

Velocity is the distance over time. It is calculated by calculating the slope of the contraction wave using the highest and lowest points.

|(DistanceA-DistanceB)/(TimeA-TimeB)| = Velocity in millimeters  per second  (2)

|(4.3-5.2)/(190-150)| = 0.0225 mm/sec (Velocity)

Period is the time it takes to complete one cycle in units of seconds.

TimeC – TimeB = Period in seconds  (3)

247-190 = 57 sec (Period)

Frequency is the inverse of period and is the number of cycles completed per second

1/Period = Frequency in Hertz (4)

1/57 = 0.018 Hz (Frequency)

Wavelength is the distance between waves and is calculated by dividing the wave velocity by the wave frequency.

Velocity/Frequency = Wavelength in millimeters  (5)

0.0225/0.018 = 1.25 mm (Wavelength)
