# Supplementary material for: Imaging the dynamics of murine uterine contractions in early pregnancy
Source: Biol Reprod. 2024 May 7;110(6):1175–90. doi: 10.1093/biolre/ioae071 (PMC11180618; doi:10.1093/biolre/ioae071)
Supplement: Legends_for_supplementary_videos_ioae071 [file legends_for_supplementary_videos_ioae071.docx]

**Legends for supplementary videos**

**Supplementary Video 1:** Contractility recording of an mTomato expressing diestrus stage uterine horn displaying contractions originating at multiple points along the uterine horn. Contractions travel both towards the oviduct and the cervix without a particular bias in one direction.

**Supplementary Video 2:** Red lines indicate borders of the uterine horn in diestrus stage as supplementary video 1. Yellow in the uterine horn indicates a contracting region and blue indicates a relaxing region.

**Supplementary Video 3:** Contractility recording of an mTomato expressing estrus stage uterine horn where a single contraction is moving towards the oviduct.

**Supplementary Video 4:** Contractility recording of an mTomato expressing estrus stage uterine horn where no directional bias towards oviduct or cervix was observed.
